# Polymeric Membranes Doped with Halloysite Nanotubes Imaged using Proton Microbeam Microscopy

**DOI:** 10.3390/nano13222970

**Published:** 2023-11-18

**Authors:** Giovanna Vasco, Valentina Arima, Soufiane Boudjelida, Mauro Carraro, Monica Bianco, Alessandra Zizzari, Elisabetta Perrone, Francesco Galiano, Alberto Figoli, Maura Cesaria

**Affiliations:** 1 CEDAD—Center of Applied Physics, Dating and Diagnostics, Cittadella della Ricerca, University of Salento, SS. 7, Km. 7300, 72100 Brindisi, Italy; giovanna.vasco@unisalento.it; 2 Department of Mathematics and Physics “Ennio De Giorgi”, University of Salento, Campus Ecotekne, 73100 Lecce, Italy; 3 CNR NANOTEC—Institute of Nanotechnology, c/o Campus Ecotekne, 73100 Lecce, Italy; monica.bianco@nanotec.cnr.it (M.B.); alessandra.zizzari@nanotec.cnr.it (A.Z.); elisabetta.perrone@nanotec.cnr.it (E.P.); 4 Department of Chemical Sciences, University of Padova, Via Marzolo 1, 35131 Padova, Italy; soufiane.boudjelida@unipd.it; 5 Department of Material Sciences, University Mohamed El Bachir El Ibrahimi, Bordj Bou Arreridj 34030, Algeria; 6 Institute on Membrane Technology (CNR-ITM), University of Padova, Via Marzolo 1, 35131 Padova, Italy; 7 Institute on Membrane Technology (CNR-ITM), Via P. Bucci 17/c, 87036 Rende, CS, Italy; f.galiano@itm.cnr.it (F.G.); a.figoli@itm.cnr.it (A.F.)

**Keywords:** halloysite nanotubes (HNTs), polyaniline (PANI), PES membranes, water permeance, proton beam probe-based imaging

## Abstract

Polymeric membranes are useful tools for water filtration processes, with their performance strongly dependent on the presence of hydrophilic dopants. In this study, polyaniline (PANI)-capped aluminosilicate (halloysite) nanotubes (HNTs) are dispersed into polyether sulfone (PES), with concentrations ranging from 0.5 to 1.5 wt%, to modify the properties of the PES membrane. Both undoped and HNT-doped PES membranes are investigated in terms of wettability (static and time-dependent contact angle), permeance, mechanical resistance, and morphology (using scanning electron microscopy (SEM)). The higher water permeance observed for the PES membranes incorporating PANI-capped HNTs is, finally, assessed and discussed vis-à-vis the real distribution of HNTs. Indeed, the imaging and characterization in terms of composition, spatial arrangement, and counting of HNTs embedded within the polymeric matrix are demonstrated using non-destructive Micro Particle Induced X-ray Emission (µ-PIXE) and Scanning Transmission Ion Microscopy (STIM) techniques. This approach not only exhibits the unique ability to detect/highlight the distribution of HNTs incorporated throughout the whole thickness of polymer membranes and provide volumetric morphological information consistent with SEM imaging, but also overcomes the limits of the most common analytical techniques exploiting electron probes. These aspects are comprehensively discussed in terms of practical analysis advantages.

## 1. Introduction

Among the versatile submicron-sized systems exploited for the preparation of composite or hybrid materials, halloysite nanotubes (HNTs) with the formula Al_2_Si_2_O_5_(OH)_4_ · nH_2_O are especially promising [1]. Their layered and tubular structure makes them similar to carbon nanotubes, but they display some advantages, such as natural availability, low cost, low cytotoxicity, and higher biocompatibility [1]. Halloysites nanotubes consist of kaolin sheets, rolled into tubular shapes, because of the lattice mismatch between adjacent layers. The external tetrahedral silicone dioxide and internal octahedral aluminium oxide provide different surfaces in terms of charge and reactivity. Indeed, HNTs exhibit the great advantage of colloidal stability and good dispersibility in water, resulting from the negatively charged surface, in the pH range of 4–9 [2]. Moreover, due to their peculiar surface chemistry, HNTs can be properly activated or functionalized on both walls and used as nano-scaffolds or nano-containers [3,4,5]. Halloysite nanotubes were exploited, for example, in catalysis [6] and photocatalysis [7,8], in biomedicine (as carriers of therapeutic compounds, for tissue engineering or medical implants [9,10,11]), for water remediation [12], and for the preparation of advanced functional materials endorsed with antifouling, self-healing, and flame-retardant characteristics [13,14,15].

Because of the external negative charge of HNTs, their properties can be modified by using polycations such as polyaniline (PANI), polyethylenimine (PEI), and polydiallyldimethylammonium chloride (PDADMAC) [16], which further increase the colloidal stability of HNTs in aqueous suspensions and provide a method to tune the release of encapsulated functional molecules [17] or increase their adsorption properties [18]. On the other hand, HNTs were used to prepare composite materials with different polymers [19], improving their mechanical and thermal properties [20].

Within this scenario, HNTs display several useful properties for the preparation of water filtration membranes. As a matter of fact, functionalized HNTs were used to support polyoxometalates (POMs) with oxygenic activity and embedded into polymeric polyether sulfone (PES) membranes as antifouling agents [21]. Due to the POM-modified surface, HNTs were shown to provide higher hydrophilicity, higher porosity, and increased permeability to the membrane [22].

Despite the deep present-day knowledge about the HNTs characterization and the development of procedures for their functionalization, the investigation on their distribution within an organic matrix using commonly available sub-micron scale imaging techniques is still challenging [23,24]. To date, the elemental characterization of HNTs has been performed by using laser induced breakdown spectroscopy (LIBS) and X-ray fluorescence (XRF) [25]. Moreover, the morphological and topographical properties of supported and free-surface HNTs distributions were investigated by using transmission and scanning electron microscopy (respectively, TEM and SEM) and atomic force microscopy (AFM) [23,26]. Nevertheless, AFM topography maps may only image samples with exposed surfaces, i.e., not embedded within a matrix material as a membrane, and conventional TEM and SEM do not allow the mapping of the composition and spatial arrangement of HNTs through the entire thickness of the membrane (hundreds of micrometers) without using cross-section configuration and thin samples, respectively. For instance, the energy dispersive spectroscopy (EDS)-based elemental mapping of ultrafiltration polysulfone membranes incorporated with varying amounts of halloysite nanotubes indicated that it may be hard to identify and localize the HNTs at low loads and in the presence of a background noise signal [27]. Moreover, even TEM-tomography, which is exploited for porosity monitoring, enables the analysis of only a small sampling volume (a few micrometers along the three dimensions), meaning there is no overview of the structure in the whole volume of the membrane [27]. Ultramicrotomy in the environmental SEM, consisting of the automated serial sectioning (thin slices of the sample with thicknesses between 50 and 100 nm) and imaging of specimens, yields the stacks of images for the subsequent 3D reconstruction of the membrane morphology [28,29]. Although larger volumes can be investigated than with TEM-tomography, with high enough resolution for pore analysis, the preparation of the samples may be critical; proper image enhancement and thresholding for 3D reconstruction may be not effective or may even be prohibitive in the presence of HNTs.

In this paper, we discuss how relevant limits can be overcome by applying nuclear microscopy (NM) to map the HNTs distribution within the membrane with their elemental information, which may be useful to control the preparation and optimize the performance of filtration membranes. In detail, we present the preparation of (1 wt%) pristine HNTs or (0.5–1.5 wt%) PANI-coated HNTs (PANI-HNTs) embedded into PES membranes and investigate the impact of HNTs on key properties for water filtration such as wettability, porosity, mechanical properties and water permeance. The experimental findings highlight the positive effect of the HNTs on permeance when they are modified with a PANI coating. In order to define the final composition of HNTs-functionalized PES membranes and image the dispersion of the HNTs inside polymeric membranes, we have exploited NM using an ion beam analysis (IBA) integrated approach, that is, a group of imaging and analytical methods based on a focused MeV proton beam that, differently from electron beams, has the capability of maintaining its directionality through the matter up to hundreds of micrometers without energy loss over longer depths. Herein, we demonstrate the unique potentiality of Micro-Proton-Induced X-ray Emission (µ-PIXE) and Scanning Transmission Ion Microscopy (STIM) using MeV proton beams for the highly sensitive detection of HNTs’ elements as well as for assessing their spatial arrangements in polymeric membranes.

To the best of our knowledge, this study presents the unprecedented application of the NM technique for the investigation of membranes embedding HNTs and, more generally, of composite systems including embedded/buried objects that cannot be imaged using standard electron-probe-based techniques for analytical and spatial mapping.

## 2. Materials and Methods

### 2.1. Materials and Sample Preparation

#### 2.1.1. Preparation of PANI-Capped HNTs

Polyether sulfone (PES) pellets, HNTs, N-methyl-2-pyrrolidone (NMP), polyethylene glycol (PEG 200), polyvinyl pyrrolidone (PVP, MW 29000), aniline, ammonium persulfate (APS), sodium phosphate monobasic monohydrate (NaH_2_PO_4_·H_2_O), sodium phosphate dibasic heptahydrate (Na_2_HPO_4_·7H_2_O), sodium chloride (NaCl), nitric acid (HNO_3_), and N,N-dimethylformamide (DMF) were purchased from Sigma-Aldrich. PANI, in the emeraldine salt (ES) form, was selected as dopant for its low-cost synthesis, flexibility, and excellent stability. The protonated emeraldine salt (ES), can be obtained through the polymerization of aniline in acidic aqueous media. For the preparation of PANI-HNTs nanocomposite, 0.5 g of HNTs was sonicated in 50 mL deionized water for 1 h. Then, APS (0.125 M) and nitric acid (0.5 M) were added, and the mixture was stirred at room temperature for 30 min. Accordingly, aniline (0.1 M) was added to the mixture to start the polymerization, which was accomplished in 3 h (see Scheme S1 in the Supporting Information). During the polymerization process, the dull mixture turns into a dark green color. The successful polymerization of PANI onto HNTs was verified using UV-VIS. The PANI-HNTs composite was collected via centrifugation in 50 mL plastic tubes and washed with deionized water (5 mL, 5 times) and methanol (5 mL, 3 times), i.e., until the surnatant became clear. The resulting HNTs capped with PANI, hereafter termed HNTs@PANI, were vacuum-dried, to obtain 0.8 g of material. The characterization was carried out using UV-VIS, attenuated total reflectance-Fourier transform infrared (ATR-FTIR), thermogravimetric analysis (TGA), TEM, and AFM.

#### 2.1.2. Preparation of Undoped and HNT-Doped PES Membranes

The PES membranes were prepared by adding three different doping systems (PANI, HNTs or HNTs@PANI). The composite membranes were prepared by dispersing 1 wt% of the dopants in NMP, under sonication, for 30 min. Then, PEG (1.05 g) and PVP (0.25 g) were added as pore-formers. PES (0.75 g) was dissolved in NMP (56.3 mL) at 60 °C for 6 h, then it was added to the same dispersion and kept under stirring at 60 °C for 2 h (with the resulting concentration in the blends being as follows: 1 wt% dopant, 21 wt% PEG, 5 wt% PVP, 15 wt% of PES).

After the complete dissolution, each mixture was sonicated for 20 min and heated without stirring for 20 min to remove trapped air. Then, the homogenous dispersion was casted (setting the casting thickness at 250 μm) onto the glass plate, for 10–20 s. After casting, the glass plate was immersed in a water bath for 1 h, then moved into another deionized water bath for 24 h and sonicated for 30 min to remove residual PVP and PEG (see Scheme S1 in the Supporting Information). The thicknesses of the resulting membranes, reported in Table 1, were measured using SEM after air-drying. The actual content of the dopants may be slightly different with respect to the nominal ones, as a result of the washing steps.

The following nomenclature will be adopted throughout the paper to refer to the membranes under study: PES is the reference PES membrane; PES + PANI (1%) is the PES membrane with the PANI additive at the concentration of 1 wt%; PES + HNTs (1%) is the PES membrane with the addition of HNTs at the concentration of 1 wt%; and PES + [HNTs@PANI] (1%) is the PES membranes with the addition of PANI-capped HNTs (HNTs@PANI) at concentration of 1 wt%. In ESI, some data are reported for PES + [HNTs@PANI] (C) with C = 0.5 and 1.5 wt% in comparison with PES + [HNTs@PANI] (1%).

#### 2.1.3. Preparation of the Samples (HNTs and PES Membranes) for NM Analysis

The new approach with the Micro NM analysis required its optimization on free HNTs before they were embedded into polymeric membranes. This step was addressed in Section S6 of the Supporting Information, where more details on the NM set-up and procedure for preparing the samples for preliminary tests are provided for the reader.

For the optimization of Micro NM analysis of HNTs, four samples were prepared via drop casting deposition of 5 µL aqueous solution of HNTs (0.83 mg/mL in PBS 50 mM, pH 7.0) on a thin (0.9 μm thick) layer of mylar (a transparent polyester film made from stretched polyethylene terephthalate) placed over a perforated aluminium plaque to be attached to the NM sample holder for µ-PIXE and FSTIM measurements.

Copper grids of 100 mesh were then applied on the sample surface to act as a spatial reference during the proton beam irradiation of the samples (Figure S1). Two membranes with embedded HNTs (PES + HNTs and PES + [HNTs@PANI] (1%), Table 1) and one membrane without HNTs (PES + PANI (1%), Table 1) were then placed on the aluminium plaques of the NM sample holder with the copper grids (Figure S1).

### 2.2. Characterization Analysis and Techniques

#### 2.2.1. Spectroscopies

The UV–VIS absorbance spectra of surnatants were recorded using a Cary 50 spectrophotometer (Varian, Palo Alto, CA, USA), with 1 cm optical path quartz cuvettes, at T = 25 °C. The ATR-FTIR spectra were obtained from solid samples using a Nicolet 5700 FT-IR instrument (Thermo Electron Corporation, Waltham, Massachusetts). Thermogravimetric analyses (TGA) of HNTs, PANI and HNTs@PANI (1%) were performed using a TGAQ500 (TA Instruments Co., New Castle, DE, USA) and recorded under N_2_ or under air, upon equilibration at 100 °C, followed by a 10 °C/min temperature ramp up to 1000 °C.

#### 2.2.2. Atomic Force Microscopy (AFM)

AFM measurements were performed on pristine HNTs and on the doped membranes. The HNTs were dissolved in phosphate-buffered saline (PBS) 50 mM, pH 7.0 (0.83 mg/mL) and deposited onto a silicon substrate. The excess of solution was removed from the substrate, the sample was then dried under a nitrogen flow and characterized using AFM (XE-100 Park instrument, Suwon, Korea) in a non-contact mode, using silicon rectangular probes (Non-Contact Cantilever PPP-NHCHR 10M, Park instrument, Suwon, Korea) with a tip radius of 10 nm. For each sample, several areas were analysed to ensure the reproducibility of the results. An analysis of the images was performed using XEI software (version 1.8.0); the dimensions of the structures here reported are, on average, over 23 objects. The uncertainties are calculated as standard deviations.

#### 2.2.3. Transmission Electron Microscopy (TEM) and Scanning Electron Microscopy (SEM)

TEM images of HNTs and HNTs@PANI samples were recorded using the 20–120 kV FEI Tecnai 12 microscope (FEI UK Ltd., Cambridge, UK) upon the deposition of aqueous dispersions (0.2 mg/mL) of functionalized HNTs onto copper grids with a continuous carbon supporting film.

Membrane samples were observed using a Zeiss, EVO MA10 SEM (Zeiss, Oberkochen, Germany) in order to investigate their morphology. The membranes were observed using a secondary electron detector in high-vacuum mode, applying an accelerating voltage of 22 kV. Cross-sections were frozen and fractured in liquid nitrogen and all samples were sputter-coated with gold prior to analysis (sputter machine Quorum Q 150R S, Quorum Technologies Ltd., Lewes, UK).).

#### 2.2.4. Micro Nuclear Microscopy for PIXE and FSTIM Analyses

A micro NM investigation (both µ-PIXE and STIM) of the samples of interest was performed using the IBA-integrated approach available at the nuclear microprobe beamline at CEDAD (Centre of Applied Physics, Dating and Diagnostics—University of Salento) [30,31], to obtain a 3 MeV energy proton beam probe focused to a spot size of 1–1.2 μm (see Section S2 in the Supporting Information for more technical details about the working principle of the NM-technique).

Micro Proton Induced X-ray Emission (µ-PIXE) is a non-destructive technique for the multi-trace elemental analysis of both organic and inorganic materials based on the detection of the characteristic X-ray emission caused by atomic inter-shell recombination events following MeV ion–atom interaction [32].

In our experiments, the µ-PIXE technique allowed us to obtain elemental information on HNTs by the detection of the characteristic X-rays emitted by their atomic constituents, that is aluminium (Al) and silicon (Si), following the interaction with a 3 MeV focused proton beam.

STIM and forward STIM (FSTIM) detect the direct and slightly deflected transmitted fraction of the protons interacting with a thin sample, respectively, and generate spatial maps by measuring energy losses or variations in the number of the transmitted particles at each pixel of the recorded image [33,34]. Nuclear microscopy experiments were conducted over several scanning areas for different magnifications with extensions ranging from 50 μm × 50 μm to 1 mm × 1 mm by using beam currents lower than 1 fA and operating for 1 h and a deflection angle of θ = 20° to acquire the FSTIM maps. The optical microscope installed in the vacuum chamber (10^−7^ mbar) allowed both to two-dimensionally translate the samples along two independent directions and to control that no damage occurred.

#### 2.2.5. Water Permeance

The water permeance tests were carried out using a dead-end filtration set-up (Amicon 8010-Millipore, Burlington, MA, USA) with a membrane area of 3.8 cm^2^. Distilled water was fed by means of a peristaltic pump (Masterflex L/S model 7518-10, Cole-Parmer Instrument Company, Vernon Hills, IL, USA) and the pressure was controlled by means of a manometer located before the filtration set-up. By varying the speed of the pump, the desired pressure was set between 0.7 and 1 bar. The permeance was calculated for each membrane under steady-state conditions (reached after about 3 h) according to the following equation (Equation (1)):

(1) $$P=\frac{m}{A\;\cdot \;t\;\cdot \;p}$$

where *P* is the permeance, *m* is the volume of permeate collected (L), *A* is the membrane area (m^2^), *t* is the operating time, and *p* the applied pressure (bar).

#### 2.2.6. Water Contact Angle and Mechanical Properties

The water contact angle (WCA) was measured using the sessile drop method employing a CAM200 instrument (KSV Instrument LTD, Espoo, Finland). For each sample, at least four measurements were taken and the average and standard deviation were calculated. The contact angle assays were repeated at different time points to evaluate the adsorption kinetics on the top surface of the membrane, corresponding to the side of the membrane in contact with the water during water permeance studies.

The mechanical properties of the membranes under consideration were measured using a Zwick Roell Z 2.5 (Zwick GmbH,. Ulm, Germany) testing unit. The membrane samples were stretched unidirectionally at a steady velocity of 5 mm min^−1^ until they broke. Mechanical properties were evaluated in terms of Young’s Modulus and elongation at break.

#### 2.2.7. Porosity

In order to estimate the porosity of the membranes, their weight was registered before and after immersion in kerosene for 24 h. The choice of kerosene is related to its lower surface tension with respect to water, which makes it more pervasive within the pores of the membrane [35]. The porosity value was then calculated using the following equation (Equation (2)) [36]:

(2) $$\mathrm{Porosity}\;(\%)=\frac{\frac{{wt}_{w-{wt}_{d}}}{\rho_{k}}}{\frac{{wt}_{w}-{wt}_{d}}{\rho_{k}}+\;\frac{{wt}_{d}}{\rho_{p}}}\times 100$$

where *w*t*_w_* is the weight of the wet membrane, *wt_d_* is the weight of the dry membrane, ρ*_k_* is the kerosene density (0.786 g/cm^3^), and ρ*_p_* is the polymer density (PES: 1.35 g/cm^3^).

## 3. Results and Discussion

This section of the paper is structured as follows. First, we present a comprehensive characterization of the membranes under study in terms of wettability, permeance, mechanical resistance, porosity and morphology. Then, through SEM inspection, the impact of HNTs on the formation kinetics and pore characteristics of the membranes is discussed, although the limitations and destructive approach of the electron-probe techniques are also highlighted. The paragraph titled “Why the nuclear microscopy technique?” provides the motivations underlying the reasoning of this study, that is the imaging and investigation limits of the conventional techniques applied to systems degradable by electron beam probing and consisting of buried objects. This passage is extremely necessary to clarify the aims of this study and give the reader further perspectives on the application of NM when dealing with the imaging and detection of buried objects by means of a non-destructive and very high-resolution approach. The valuable comparison between the information provided by micro-PIXE and FSTIM maps and other well-known techniques (AFM and SEM) clarifies the unique potentialities of the NM technique in the investigation of embedded objects.

### 3.1. PANI-Coated HNTs

As outlined in the Experimental section, the coating of HNTs with PANI was performed via the direct polymerization of aniline onto the HNTs, and the conditions were selected to foster the formation of the emeraldine salt (ES) of PANI. In this form, indeed, PANI is conductive and more hydrophilic. The PANI coating is advantageous because it increases the stability of the HNTs’ dispersion in polar solvents. In this respect, the polymerization of PANI onto HNTs was assessed using UV-VIS upon the dispersion of the sample in DMF (Figure S2) [37]. The occurrence of the peak at 800 nm, assigned to benzenoid–quinoid rings’ charge transfer, confirmed the formation of the ES form of polyaniline.

**Figure 1:**
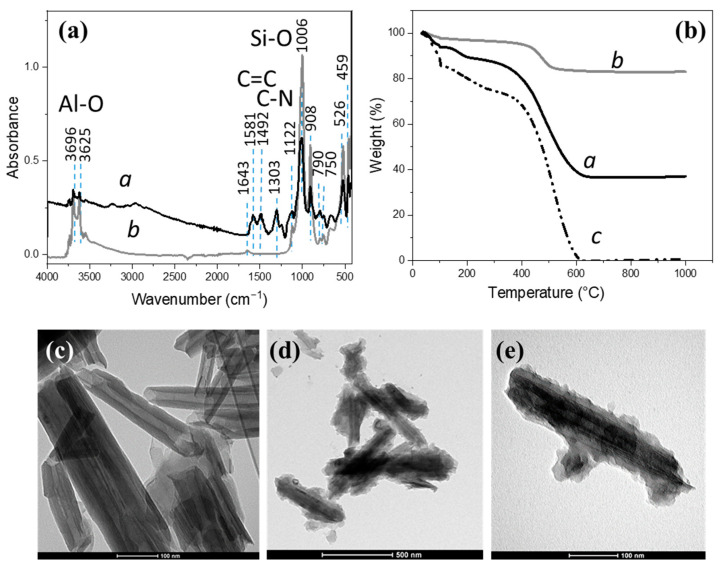
(**a**) ATRFTIR spectra of HNTs (*a*) and of HNTs@PANI (*b*) core-shell structures. (**b**) TGA of HNTs (*a*), HNTs@PANI (*b*), and PANI (*c*), obtained under air, from 100 °C to 1000 °C, with a 10 °C/min ramp. TEM micrographs of pristine HNTs (**c**) and HNTs@PANI (**d**,**e**) from an aqueous dispersion.

ATR-FTIR data are shown in Figure 1a and allow to identify the functional groups characteristic of both involved materials. The region from 1200 to 1650 cm^−1^ exhibits strong signals that are ascribed to PANI and, in particular, the bands appearing at 1250–1600 cm^−1^ are assigned to the vibration of quinoid rings (“N=Q=N”) and benzenoid rings (“N-B-N”). The signals of HNTs can be observed in the range 820–1650 and 450–600 cm^−1^. The bands observable at 3696 and 3625 cm^−1^ can be assigned to the O-H stretching vibration of the Al-OH groups of the internal surface and inner Al-OH groups, respectively. The peak at 908 cm^−1^ is assigned to the deformation of inner interfacial Al-OH groups.

The strong band centered at 1006 cm^−1^ corresponds to the in-plane Si-O stretching vibrations, while the peak situated at 790 cm^−1^ corresponds to the symmetric stretching of Si–O-Si and the peak at 750 cm^−1^ is related to perpendicular Si–O-Al stretching. The peaks at 526 cm^−1^ and 459 cm^−1^ likely correspond to the bending vibrations of Si-O-Al and Si-O-Si bonds, respectively [38]. In the HNTs@PANI derivative, the most intense peak is split at 1008 and 1024 cm^−1^, in agreement with the occurrence of an interaction between HNTs surface and PANI.

TGA analysis was performed to assess the thermal stability of the nanostructures as well as their composition in terms of organic coating with respect to the inorganic domain. From the TGA plots in Figure 1b, a major weight loss can be observed between 500 and 950 °C. The degradation step of HNTs before 350 °C is related to the evaporation of the water both adsorbed on the surface and within the interlayer between the SiO_2_ and Al_2_O_3_ sheets, while a dehydroxylation occurs at 400–550 °C [38,39]. The thermal degradation of the PANI (between 400 and 600 °C) is ascribed to the decomposition of the polymer chains. In the case of HNTs@PANI, the thermal stability improves with respect to PANI, due to a barrier effect favored by the interfacial interaction between the polymer and the HNT [40].

From a comparison of the samples with and without PANI (52 wt% and 82 wt% residual weight, respectively, at 1000 °C), we can calculate the contribution of the organic coating in the composite, corresponding to about 30 wt%. Furthermore, a TEM analysis of the HNTs@PANI samples in Figure 1c shows the rod-like shape of the HNTs under consideration and the presence of the PANI coating that can be clearly distinguished around all the isolated tubes as an irregular but continuous layer with an average thickness of about 20 nm.

### 3.2. Surface and Bulk Properties of Undoped and Doped PES Membranes

PES membranes were prepared as detailed in the experimental section. The addition of different additives determines changes in the bulk properties of the membranes. As the most significant result, with a clear applicative outcome, membranes become more water-permeable with 1% of HNTs@PANI (Table 1).

In the literature, membranes with high hydrophilicity and high porosity are reported to be more water-permeable than hydrophobic and low porosity ones [41]. We have performed some studies based on a combination of conventional techniques (ATR-FTIR, contact angle, porosity and mechanical properties, SEM) and the STIM approach to investigate the reasons of the higher efficiency of PES + [HNTs@PANI] membranes.

As a first compositional study, the ATR-FTIR spectra of the membranes were acquired. The study demonstrates that the superficial composition of the membranes is basically unaffected by the small content of dopants and shows the expected signals pattern of the PES matrix (Figure S3) [39,40,42]. This could be due to a possible preferential localization of the dopants underneath and inside the membrane rather than on the surface.

Then, we evaluated the surface wettability of the three membranes via WCA, both in static and time-dependent conditions. Time-dependent WCA measurements are able to determine the surface water absorption kinetics (see Figure S4) and are quite different from water permeance studies. Indeed, while permeance experiments evaluate the water released from the membrane under pressure-induced compaction, the time-resolved WCA measurements are related to the spontaneous wetting and absorption process of a calibrated water droplet deposited on the top of the membrane (from the filtration side).

In general, a decrease in the WCA value is associated with the occurrence of hydrophilic additives improving the water uptake/hydrophilicity of PES, once they distribute closely to the top surface of the membrane. For instance, the presence of protonated-NH-groups might imply an increase in the Van der Waal’s interactions and hydrogen bonding of HNTs@PANI with water molecules, hence improving the hydrophilicity and penetration ability of water. In our experiments, static WCAs, ranging from 68 to 73°, show a minimal reduction on turning from PES to PES + PANI and PES + HNTs and no relevant impact when adding HNTs@PANI, even at different loads (see data in Table S1). As already observed via ATR-FTIR, the presence of dopants has a small or negligible effect on the surface features, which are similar to that of PES, in agreement with the good wettability observed for all the membranes (see Table 1).

The hypothesis that additives have a limited effect on surface properties but may somehow affect the water adsorption properties is supported by the time-dependent WCA measurements as a function of the additive reported in Figure S4. PES + PANI (1%) shows the strongest variation in WCA over the explored range, up to an almost complete absorption of the droplet after 140 s (indicated by the blue star in Figure S4a), pointing to a fast decrease in the WCA to 0° [43]. The amount of dispersed additives also influences the absorption kinetics, as observed for membranes at different HNTs@PANI loads (see Figure S4), suggesting that the different process of water uptake promoted by the incorporation of these dopants in PES membranes is poorly mediated by superficial effects.

Hence, in the absence of any evident correlations between the surface hydrophilicity and the increased water permeance observed with the addition of HNTs@PANI, we focused on the morphological features of membranes such as thickness, porosity, pore size, and mechanical properties.

Although all membranes were prepared using a casting device set at the same height (250 μm), a strong impact results from the presence of the dopants. The thickness of the membrane samples was found to be 50 µm for a PES pristine membrane and ranged from 80 to 129 µm for membranes loaded with HNTs and PANI (Table 1). In all cases, the overall thickness increases, with larger values for HNTs@PANI dopants.

As already observed in the literature [44], the addition of HNTs (PES + HNTs membrane) leads to an increase in the membrane pore size (from 0.14 μm to 0.74 μm), as a consequence of the fact that HNTs are hydrophilic agents which can increase the demixing rate between solvents and non-solvents (water) during the membrane formation. A similar effect, although to a lesser extent, is observed for the addition of PANI, which results in an increase in membrane pore size (0.52 μm) as a consequence of PANI’s hydrophilic nature [44].

The simultaneous addition of HNTs and PANI resulted in a further increase in pore size (Table 1 and Table S1) with respect to the pristine PES membrane and to the membranes prepared with only PANI and HNTs.

The mechanical properties of the membranes, in terms of Young’s Modulus (Table 1 and Table S1), seem to be related to the porosity. As generally observed in the literature, the Young’s Modulus appears lower for the higher porosity values. The increase in the overall void fraction is responsible, in fact, for a decrease in the membranes’ mechanical properties, since voids represent weak points in the polymer matrix [45]. The highest value of Young’s Modulus (54 MPa), in fact, was measured for the membrane PES + PANI as a consequence of its lowest porosity degree (86%). The addition of HNTs, on the contrary, led to a decrease in the membranes’ Young’s Modulus due to the increase in membrane porosity.

The elongation at break, which is the ratio between the changed length and original length after the fracture of the membrane, displayed small values for all the membranes evaluated (between 4 and 9%). As shown in Table 1, the addition of dopants (PANI and/or HNTs) did not compromise the elasticity of the membranes in comparison to the PES pristine one. The measured values of Young’s Modulus and elongation at break are comparable to the common values generally measured for PES membranes [46].

While the increased pore size of the PES + PANI and PES + HNT membranes does not coincide with a water permeance improvement, the membrane prepared with PANI@HNTs shows larger pores and results in more permeability than the other membranes, highlighting the synergistic effect of the dopants (Table 1). A likely hypothesis on the beneficial effect of PANI coating is that it can tune the strong interfacial interactions between HNTs and PES [47], leading to an improved morphology with larger and interconnected pores. Interestingly, within the series reported in Table S1, it is evident that, by decreasing the content of PANI@HNTs additive, both the overall porosity and the permeance increased, with optimal results at 0.5 wt%.

Notably, the permeance was not stable but decreased over time for all samples. One of the explanations of the permeance data can be the compaction phenomenon which occurs during the testing experiments. In a previous work, Kamal et al. [48] already observed that polysulfone membranes suffered from a considerable compaction when subjected to hydraulic pressure, which led to a drastic decrease in water permeance. The addition of HNTs at low concentrations (<0.5 wt%), on the contrary, resulted in more permeable membranes, also owing to the reinforced porous polymer structure.

The scarce correlation between permeance and porosity data for the different dopants suggests that further and more detailed morphological studies are needed to explain the different permeance behaviours. Indeed, a possible reasoning could be that pores with different shapes, distributions, and connections among themselves and the membrane surfaces are originated during the membrane development and assembly processes leading to a complex morphology which varies as a function of depth. In general, whereas the skin layer is directly in contact with the fluid to be filtered and onsets the filtration process, a role is played by the underlying porous layer and the structure/morphology of the pores connected to the skin layer and to the large pores settled on the backside of the membrane [49]. To favor the filtration process effectively, pores connected with the membrane surface and going through the skin layer need to be connected with the bottom surface of the membrane and to avoid clogging under compaction. Hence, a characterization that is able to provide information on the pore characteristics as a function of the whole depth at a high enough spatial resolution is critical for a detailed understanding of the permeance data. To this aim, SEM and NM analyses were performed and compared, as discussed in the next paragraphs.

### 3.3. SEM Investigation of Undoped and Doped PES Membranes

**Figure 2:**
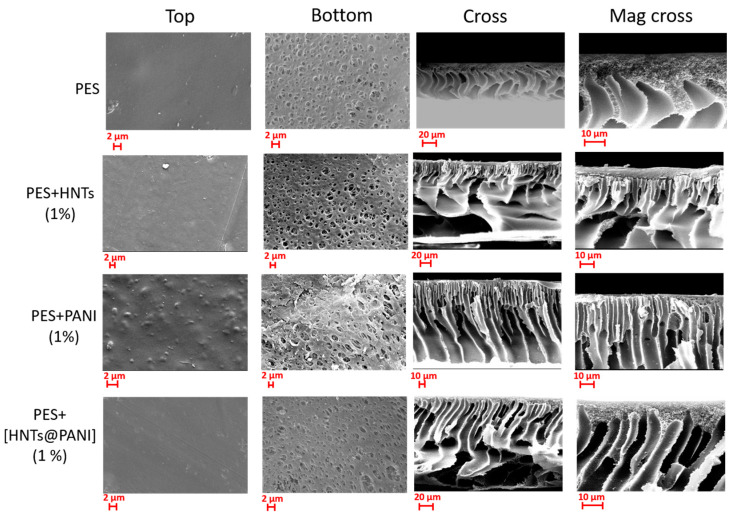
SEM morphology of the investigated membranes, as labelled on the left column. From the **left** to the **right**, the top surface, bottom surface, cross-section, and magnified cross-section are shown.

Morphological observations of the porous structures were conducted on the top surface, bottom surface, and cross-section of the membranes under study, as a function of kind (PANI, HNT, and HNTs@PANI) and HNTs@PANI additive dosage. The associated SEM images (Figure 2 and Figure S5) show that all samples exhibit the typical morphology of membranes prepared via a phase inversion procedure [50], that is an asymmetric structure consisting of a dense top layer (the so-called skin layer) supported by a porous region (bottom) with fully developed macro-pores featuring aligned or distorted finger-like channels along the cross-section and a porous bottom surface.

The observed morphology can be discussed on the basis of the kinetic and thermodynamic aspects occurring during the phase inversion process [51]. Membranes characterized by a finger-like or macrovoid structure, like the ones prepared in this study, are generally the result of an instantaneous demixing rate occurring during the formation phase [50,51,52]. The presence of several hydrophilic agents, such as PVP, PEG, PANI, and HNTs, used in the preparation of the membranes, favoured the exchange between solvent (NMP) and non-solvent (water), hence accelerating the demixing rate of the cast film and leading to the formation of membranes with a compact top layer and a macrovoid/finger-like structure along the cross-section [53]. The addition of HNTs and PANI, in general, resulted in the formation of two distinct morphologies visible along the cross-sections: an upper structure made of straight, short finger-like channels just beneath the top skin layer and a lower structure characterized by larger and elongated pores (e.g., macrovoids) extending to the bottom of the membrane. The addition of HNTs to the PES membrane led to the suppression of its sponge-like structure, visible just beneath the top layer, in favor of narrow and short channels supported by a very porous region characterized by macrovoids. A similar morphology was also observed by Kamal et al. as a consequence of the incorporation of HNTs into polysulfone-based membranes [54]. This phenomenon was mainly ascribed to the increased thermodynamic instability of the membrane system upon the addition of HNTs (above 0.5 wt%) which accelerated the solvent/non-solvent exchange rate. The presence of PANI as an additive, in the case of a PES + PANI membrane, resulted in a cross-sectional structure characterized by a higher number of longer and vertical finger-like pores widening in the bottom layer. In this regard, Subtil et al. [55] observed the same type of structure for membranes prepared in PES and PANI.

The morphology of the membrane PES + [HNTs@PANI] is a combination of the structures observed before being characterized, along the cross-section, by a thin sponge-like sub-structure, a finger-like middle morphology, and a bottom macrovoid structure. SEM cross sections would suggest that the more regularly aligned finger-like structures ending with sponge-like large features and voids toward the support-facing side of the membrane, as exhibited by PES + [HNTs@PANI], could be responsible for the improved permeance due to their channeling of water across the membrane thickness effectively and releasing it quickly on the bottom side. Notably, they appear to be directly connected to the upper surface through the skin layer that is thinner for PES + [HNTs@PANI] than for the other membranes.

### 3.4. Proton-Probe-Based Investigation of Doped PES Membranes

#### 3.4.1. Why the Nuclear Microscopy Technique?

A few preliminary remarks are worth mentioning to rationalize the choice of NM, rather than the common imaging techniques in material science, to spatially localize HNTs incorporated in polymer membranes.

Atomic force microscopy (AFM) is particularly suitable for the 3D high-resolution topographic imaging of surface structures without damaging effects, due to its preliminary sample treatments and high-vacuum conditions. Disadvantageously, it does not allow the scanning of large areas on rough substrates, because it is limited to tens of micrometres in the Z-scan range and to a maximum 100 µm × 100 µm scan area in the X and Y directions. Hence, a topography analysis of areas extending over several cm^2^ using AFM can be time-consuming and unpractical due to the necessity of multiple acquisitions at a relatively slow scanning rate. Additionally, and mainly, AFM is unable to image buried objects, meaning it is not a suitable investigation tool for HNTs incorporated in a polymer matrix.

The elemental information and imaging of many hundreds of microns extended areas are enabled by SEM equipped with EDS, which can be combined with AFM for studying sub-micron sized objects [56,57]. However, SEM usually requires the sample to be kept under a vacuum, as well as a treatment with conductive materials or a conductive coating in the case of insulating samples to prevent electron charge accumulation. Transmission electron microscopy (TEM), which also provides structural information but at much higher resolution than SEM, may be unworkable, because the extraction of transmitted electrons introduces limits on the thickness of the samples [57], and it is disadvantageous with respect to SEM due to its high-vacuum working conditions and very expensive equipment [58,59].

Advanced methods for morphological 3D characterization, such as TEM tomography and ultramicrotomy in the environmental SEM, allow for the sampling of small volumes leading to no realistic overview of the whole membrane structure [27]. The automated serial sectioning of the sample in thin (50–100 nm thick) slices and their imaging require the subsequent 3D digital reconstruction of the membrane morphology [28,29]. Hence, in the presence of HNTs additives, the preparation of the samples can be even more critical and 3D reconstruction via digital image processing is unworkable.

Furthermore, as the electron probe can induce severe damages on polymers (chain scission, ionisation, and breaking chemical bonds) [60,61], the electron microscopy analysis of polymeric materials can be applied by setting specific energetic parameters, low-vacuum conditions, and chemical treatments, which are not suitable for the contemporary investigation of embedded NPs [58,59].

Unlike the above discussed approaches, NM can also be performed on untreated samples, without the requirement of chemical fixation or cryofixation (for biological samples) and allows the acquisition of elemental maps of the characteristic X-rays emitted, from micrometric to millimetric squared [34,62,63,64,65]. In terms of sensitivity level, a proton probe can detect elemental traces at a resolution of some parts per million [66], which is well beyond the sensitivity limits of a scanning electron microprobe (one part in 10^3^). Other advantages of NM are that MeV proton beams go through matter along defined straight paths by keeping their focusing degree, with low scattering and a deep linear energy transfer. Additionally, NM refers to a group of several Ion Beam Analysis (IBA) techniques exploiting a proton beam that allows for the acquisition of different kinds of information in a single working session and at a higher sensitivity than energy-dispersive X ray spectrometry (SEM-EDS) [67,68,69]. To date, despite the low cost and automated procedure of EDS elemental analysis, the discrimination of residues and presence of contaminants may not be detectable due to the sensitivity limits of the technique (bremsstrahlung, background effects). Although elemental analysis is also provided by X-ray photoelectron spectroscopy (XPS), with the advantage of obtaining information about the bonding state, XPS is a surface technique with a sampling depth of up to 10 nm. On the other hand, bulk information via sputtering methods is not only invasive and destructive but also leads to not-so-accurate data in the case of polymers [66,70]. Moreover, molecular structure characterization, performed by means of micro-Fourier Transform Infrared Spectroscopy (FTIR), may suffer from low signal-to-noise gain and micrometer-scale depth analysis [71].

On this basis, we exploit a proton micro-beam probe for studying HNT-doped polymer ultrafiltration membranes and demonstrate, in practice, a few important points that make this approach not an alternative technique, but one that is valuable and unique in some aspects, for investigating the spatial and compositional distributions of sub-micron sized HNTs.

To begin, a distribution of HNTs imaged via AFM analysis enables a first look at the possibilities offered by NM and its strong points. Among the common techniques, AFM can be exploited to investigate the morphology, average size, and surface features of un-embedded HNTs because of its applicability to a wide range of resolutions (from sub-micron to micron scales), the lack of preparation requirements for the sample, and the reduced cost with respect to electron microscopy set-ups [72]. However, as detailed in the Supporting Information (Section S6 “Comparison between AFM and NM investigation of HNTs”), our preliminary AFM-based investigation, based on two-dimensional AFM topographies of HNTs distributions acquired over areas of 2 μm × 2 μm up to 30 μm × 30 μm (see Figures S6 and S7a), demonstrates partial information restricted to relatively small areas, suspicious morphology over larger areas due to point-induced artefacts, and no systematic elemental/compositional information. Upon comparing AFM and micro-PIXE maps, it is found that these techniques show a good agreement in their imaging issues when inspecting a 30 μm × 30 μm scan area (Figure S7a,b). Notably, while the AFM scanner technology limits the imaging of areas larger than 30 μm × 30 μm, micro-PIXE maps can be acquired over areas up to 1 mm × 1 mm (Figure S6d) without acquiring multiple images and with a high resolution (some parts per million).Therefore, upon comparing NM and AFM, several advantages of the NM technique are clearly demonstrated: in (i) elemental information and (ii) the imaging of the HNTs over areas comparable to the ones accessible using AFM without the drawbacks of AFM and, in addition, over areas, of the order of mm^2^, that are not accessible using AFM and electron microscopy in a single scan.

The morphology of the HNTs is not detectable by using either the µ-PIXE or the FSTIM maps. This issue is expected due to the relationship between the minimum achievable size of the focused proton beam (~1 µm) and the sub-micron size of HNTs. However, it is not a limit in case of distribution studies. As the proton probe can identify the HNTs throughout the detection of aluminium and silicon constituents, the high sensitivity of the µ-PIXE technique can provide the spatial arrangement of HNTs with high detection sensitivity. On the other hand, μ-PIXE overperforms other common techniques for acquiring compositional maps over areas, even of the order of mm^2^, that allow the identification of the general characteristics of the distribution in real samples and create statistics relating to a region of interest without multiple time-consuming acquisitions. Nuclear microscopy not only detects the presence of HNTs based on the elemental identification of their constituents, but can also discriminate residues and contaminants with high sensitivity. Moreover, thanks to a complementary IBA approach, when analysing a selected area using μ-PIXE, it is possible to associate the related in-depth analysis of the polymeric matrix with the same area applying FSTIM. So, the μ-PIXE and FSTIM maps, which are 2D projection images of the in-depth 3D information, must be read by the operator in an integrated way.

The potentialities of the NM technique are particularly remarkable If HNTs are incorporated in a polymer matrix because, in this situation, while AFM may only image surface and partially uncovered HNTs, a proton beam can also probe buried HNTs. Moreover, as large areas (from hundreds of squared microns to squared millimetres) can be scanned using a proton beam, the resulting spatial maps are representative of the distributions of HNTs in the membranes. In fact, a MeV proton beam can penetrate the matter maintaining a straight path with negligible deviation over hundreds of micrometres [73,74,75,76,77,78].

Notably, for an aromatic polymer such as PES, protons beams were found to be responsible for less degradation than electron beams [79]. Also, PES was observed to be structurally resistant to the irradiation of protons for beam fluence up to 10^14^ ions/cm^2^ [78].

Despite SRIM (Stopping and Range of Ions in Matter) simulations indicating that a 3 MeV proton beam can penetrate up to 115 μm in PES bulk samples [78], the considerable percentage of the porosity of the membranes allows the proton beam to travel beyond such a stopping range estimation. The detection of FSTIM maps in our experiments demonstrates the protons transmitted after travelling the whole thickness of the membranes.

#### 3.4.2. PANI- and HNT-Doped Membranes Investigated using+ the NM Technique

Polymer membranes embedding the HNTs were studied by means of the NM method over scanning areas extending from 50 μm × 50 μm up to 1 mm × 1 mm.

**Figure 3:**
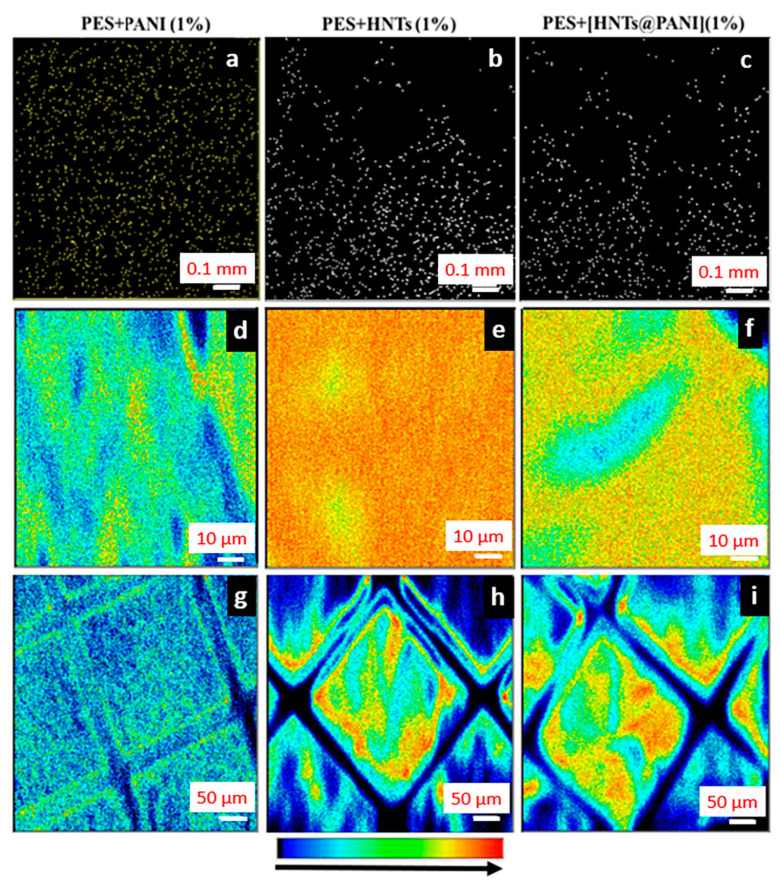
µ-PIXE maps over a scanning area of 1 mm × 1 mm of the (**a**) sulfur (S) signal (a yellow signal on a black background) in the PES + PANI (1%) membrane, and of HNTs ((**b**,**c**) with a white signal from the combined detection of aluminium (Al) and silicon (Si) on a black background) in the PES + HNTs (1%) and PES + [HNTs@PANI] (1%) membranes. FSTIM maps acquired over 100 µm × 100 µm of (**d**) PES + PANI (1%), (**e**) PES + HNTs (1%), and (**f**) PES + [HNTs@PANI](1%). FSTIM maps acquired over 500 µm × 500 µm of (**g**) PES + PANI (1%), (**h**) PES + HNTs (1%), and (**i**) PES + [HNTs@PANI] (1%).

Figure 3 presents the NM analysis of PES + PANI, working as the reference sample, as well as the membranes PES + HNTs (1%) and PES + [HNTs@PANI] (1%). Figure 3a reports the μ-PIXE maps, acquired over a 1 mm × 1 mm area, of sulphur (S) that tracks the presence of PES for the PES + PANI sample. Since light elements (i.e., hydrogen, carbon, nitrogen, and oxygen) constituting PES and PANI cannot be detected using μ-PIXE, Figure 3b,c report the cumulative silicon (Si) and aluminium (Al) signals associated with HNTs, for the membranes PES + HNTs (1%) and PES + [HNTs@PANI] (1%), respectively.

As follows, a statistical analysis of the µ-PIXE maps performed using the open-source ImageJsoftware (version 1.53s) allowed us to identify HNTs and estimate the percentage of HNTs (in volume) in the case of the membranes PES + HNTs (1%) and PES + [HNTs@PANI] (1%) (Table 2).

Notably, µ-PIXE maps are informative regarding the amount of HNTs within the volume of the membrane, because a two-dimensional µ-PIXE map is a plane-view projection of the composition within the whole measured volume. Accounting for the µ-PIXE maps acquired over areas of 50 µm × 50 µm, 100 µm × 100 µm, 500 µm × 500 µm, and 1 mm × 1 mm, the percentage of HNTs was found to be almost independent of the scanning area and slightly decreasing on turning from PES + HNTs (1%) to PES + [HNTs@PANI] (1%), consistent with the fact that the contribution of the organic PANI domain is about 30 wt% in PES + [HNTs@PANI] (1%).

The independence of the calculated HNTs percentage on the scan area indicates the homogenous dispersion of the HNTs in the entire volume of the membrane.

The dark regions can be ascribed to the proton beam crossing macrovoid regions, meaning the absence of interactions with the target HNT. Therefore, differences in the depth distribution and structure of macrovoids result in different arrangements and densities of voids in the NM maps.

Further, an estimation of the density of HNTs (number of HNTs per unit volume) was obtained, accounting for the µ-PIXE maps. Once the volume was calculated through the product of the area over which the µ-PIXE map was acquired (i.e., 1 mm × 1 mm) and the thickness of the membrane (80 µm for PES + HNTs (1%) and 129 µm for PES + [HNTs@PANI] (1%)), the measured porosity was exploited to subtract the void volume from the total volume and effectively fill the volume with HNTs and polymer matrix. The number density of HNTs resulting from dividing the HNT counting using ImageJ [80] and the effectively filled volume was found to yield a load of HNTs larger for PES + HNTs (1%) than for PES + [HNTs@PANI] (1%) (Table 2). This estimation further confirms that the real amount of HNTs in PES + HNTs (1%) is higher than in PES + [HNTs@PANI] (1%) due to the presence of the PANI-capping layer.

After having checked the HNTs presence and their distribution using µ-PIXE, the contemporary acquisition of FSTIM maps, obtained from the detection of the transmitted protons (θ=20°), enabled us to verify the membrane structure through proton scattering. Indeed, the signal of the HNTs obtained from µ-PIXE maps is expected to reflect the distribution of voids and finger-like channels in that the proton interaction depends on the effective volume of crossed material and the distribution of pores in terms of their projection on the bottom surface, obtained from the FSTIM maps.

**Figure 4:**
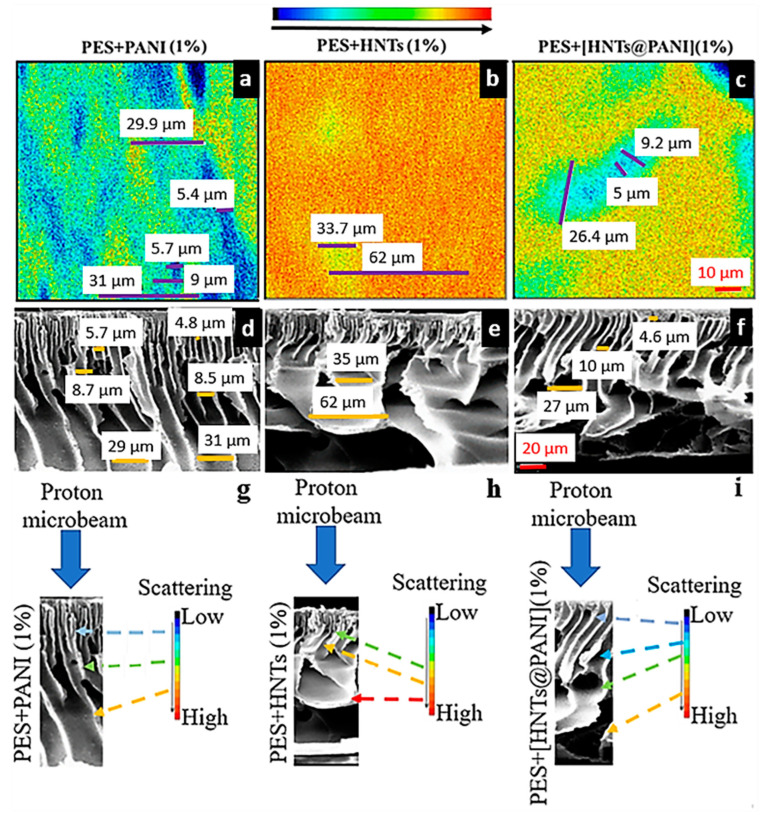
(**a**–**c**) FSTIM maps (top row) on a scanning area of 50 μm × 50 μm of (**a**) PES + PANI (1%), (**b**) PES + HNTs (1%), and (**c**) PES + [HNTs@PANI] (1%). (**d**–**f**) The corresponding SEM scans of the cross sections (central row) and (**g**–**i**) a scheme showing the relationship between the information from the SEM cross section and the degree of proton scattering induced by the structural properties (size and morphology of voids) of the membranes.

On turning to the FSTIM area density maps acquired over the 100 µm × 100 µm and 1 mm × 1 mm scan areas of all the samples under consideration (Figure 3d–i), the density variation (represented by a colour variation from blue to red, according to the colour bar scale in Figure 3) can be related to the occurrence of pores. Generally, since the proton scattering cross section increases as the squared atomic number of the target, a higher probability of scattering from the HNTs (aluminium and silicon) than the constituents of the polymeric membrane (oxygen, carbon, hydrogen, sulphur) results. Considering the configuration of the photodiode detector at θ=20° with respect to the incident beam direction and scattering enhancing, going from dark to red in the color bar scale dark-blue areas in the FSTIM maps, means the incoming protons are directly transmitted throughout the material or the sample does not induce any significative change in the protons’ trajectory. Therefore, the presence of HNTs determines a considerably lower presence of dark-blue areas at very low scattering compared to the control sample PES + PANI (1%) (Figure 3d). Indeed, a turn towards red color in PES + HNTs (1%) (Figure 3e,f) suggests a higher percentage of HNTs with respect to PES + [HNTs@PANI] (1%), as reported in Table 2. Nevertheless, protons are also scattered by the structural characteristics of the membranes such as their macrovoids and/or finger-like pores. Hence, FSTIM maps can advantageously and especially be exploited to gain morphological information about the membranes. For a full understanding of this aspect, the FTIM maps were compared with SEM images in Figure 4.

#### 3.4.3. Correlation between FSTIM and SEM Mapping of PANI- and HNT-Doped Membranes

Figure 4, which compares the FSTIM maps and SEM cross sections of the membranes under consideration, is meaningful for the correct interpretation of the FSTIM maps and demonstrates the predictive power of FSTIM in terms of picturing the structure of the membranes. Figure 4g–i draws the relationship between the information from the SEM cross section and the degree of proton scattering induced by the structural properties (size and morphology of voids) of the membranes.

To obtain a correct association with the SEM cross-sections, it is worth observing that a 2D FSTIM map provides the volumetric information that results from the in-plane projection of the arrangement and morphology of voids within the depth of the membrane. The association of the different colours with the structures, also considering the proton scattering process, is a necessary step to set an applicative protocol. In fact, on proceeding along the depth, the presence of the voids yields a much weaker interaction and deviation of the proton beam from the membrane. Proton scattering along small angles is no longer imaged with blue colour (meaning very low scattering), but with green and yellow colours, depending on the overlap between the projection of voids and filled parts of the membrane contributing to proton scattering.

Therefore, any change in the density of the filled part of the membrane that affects the weight of the scattering implies a different colour on the scattering scale bar. For instance, the plane projections of the cross-sectional structures yield narrower and shorter regions associated with voids in the case of PES + HNTs (1%) than in the case of PES + [HNTs@PANI] (1%). This issue can be due to the presence of less elongated and twisted finger-like voids in the former case. That is, for PES + PANI (1%), the presence of more vertical channels, which allowed the protons to follow a generally straight pathway through the membrane, is reflected in the FSTIM map (Figure 4g); the blue areas, associated with the detection of weakly scattering protons, correspond to the diameter of the channels beneath the skin layer. Their diameter, ranging between 5.4 and 5.7 µm, is comparable to those revealed in the SEM image (4.8–5.7 µm). While interacting with the whole thickness of the membrane, the beam undergoes weak scattering contribution due to the interception with the walls of the enlarging voids. This interaction is imaged with green-yellow areas, which correspond, respectively, to the expansion of the voids in the middle of the membrane and their widening in the final section in depth (Figure 4g). Their diameters, enlarging from 9 µm up to 29.9–31 µm, correspond to the finger-like structure in the SEM image (respectively, 8.5–8.7 µm and 29.7–31 µm) (Figure 4a,d). It should be noted that the FSTIM measurements of the void’s dimensions cannot be related to the pore size analyses (Table 1). Pore size measurement, in fact, refers to the minimum dimension (or width) between two opposing walls in a pore, placing, in this case, all the studied membranes in the microfiltration range.

The occurrence of macrovoids connected to the surface by a thin skin layer and developing more vertically and regularly along the thickness of the membranes PES + HNTs (1%) and PES + [HNTs@PANI] (1%) are expected to result in regions associated with low proton scattering (blue and bluish colours) and different degrees of scattering (colour turning in green-yellow to orange areas) over the projected 2D map, depending on the effectively crossed proton path within the filled regions of the membrane embedding HNTs. Upon comparing the FSTIM and SEM images, it can be argued that both PES + HNTs (1%) and PES + [HNTs@PANI] (1%) show red areas where protons suffered from enhanced scattering with respect to PES + PANI, due to the HNTs dispersed in the polymeric matrix (Figure 4b,c). Anyway, the high scattering of the protons in PES + HNTs (1%) is also induced by the small diameters of the channels just beneath the skin layer and the enlargement of the channels that progressively change their directions, being horizontally projected, so that the red colour is dominant in the map (Figure 4b,h). However, the yellow areas (33.7 µm in diameter), where the protons are less scattered, correspond to the enlarging voids at the beginning of the projections, comparable to the 35 µm of the SEM image (Figure 4b,e,h). Moreover, the yellow undertones allow the identification of the enlargement of the underlying projections, estimated to be 62 µm both in the FSTIM and SEM images (Figure 4b,e,h). On the contrary, the FSTIM map of PES + [HNTs@PANI] (1%) shows elongated areas, where the thickness of the central zone imaged in blue (5 µm in diameter) corresponds to the finger-like channels beneath the skin layer (4.6 µm in the relative SEM image) that did not cause relevant proton scattering events (Figure 4c,f,i). Within the finger-like voids enlarging in depth, a light blue contour arises, whose diameter reaches 9.2 µm (compatible with the 10 µm spotted in the SEM image). The extension up to 26.4 µm in the same FSTIM image is consistent with the 27 µm enlargement in the SEM image corresponding the changing orientation of the projection (Figure 4c,f,i), turning along a green area. Then, the interaction with the final horizontal projections induces protons to be highly scattered, thus resulting in a yellow-red background (Figure 4c).

Definitively, colour association with the scattering process in FSTIM maps constitutes key information in understanding and individuating the volumetric and morphological contribution of the cross-sectional structures (voids and filled parts embedding HNTs) developing though the membranes.

A final remark concerns the permeance data, that the above morphological analysis, in terms of degree of proton scattering, enables us to explain as follows. The SEM cross sections of PES + [HNTs@PANI] would suggest that more regularly aligned finger-like structures, well connected with a thin skin layer and ending with sponge-like large features and voids toward the support-facing side of the membrane, could be responsible for the improved permeance, due to their ability to channel water across the membrane thickness effectively and releasing it quickly on the bottom side. In addition to the visual inspection of morphology facilitated using SEM, differences in the diffusional exchange rates stemming from the effective diameter and overall number of pores can be relevant to discussions of the permeation performance in porous filtration membranes. In this respect, FTIM maps allow for estimating the relative contribution of the void to the filled parts embedding HNTs, as well as the finger-to-sponge structure through the colour scale bar. For instance, the FTIM maps associated with PES + HNTs and PES + [HNTs@PANI] make clear that the proton beam has lower scattering while travelling PES + [HNTs@PANI]. Hence, as the colour maps are 2D projections indicative of the effective weight of voids through proton scattering, closed (lacy) structures as well as coarse (regular and aligned) distributions are associated with large (small) scattering and reduced (increased) permeance.

The above discussion clearly points out that analyses yielding information on average pore size and distribution, such as porosity and cross section SEM images, are not able to provide a detailed characterization of how the inner structure and the void-to-filling ratio affects the functional permeance. On the contrary, characterization using the NM technique enables us to gain deeper insight into the interplay between the compositional/morphological properties and functional performances of membranes embedding HNTs via a proper interpretation of the proton scattering signal.

## 4. Conclusions

PES membranes for water filtration were doped either with PANI, uncapped HNTs or PANI-capped HNTs at a 1% wt concentration. With respect to the PES blank membrane, the dopants have an effect on membrane morphology (thickness, pore size, and shape). However, only when the two dopants, HNTs and PANI, are combined, the membranes display enhanced water permeance.

The permeance results are not explained in terms of an increase in surface wettability since the WCA values do not change significantly. All the membranes, indeed, display a hydrophilic nature as a consequence of the hydrophilic nature of PES and of the dopants employed.

On the other hand, measurements of WCA evolution over time have been interpreted as mainly reflecting the surface properties as correlated to the spontaneous water absorption by the bulk membrane.

These aspects have pointed out the importance of an in-depth morphological investigation that has been carried out via a comparison between conventional SEM cross-sectional analysis and combined μ-PIXE with FSTIM techniques.

SEM micrographs have shown that the morphology of the manufactured membranes is characterized by a compact skin top layer and a porous sublayer consisting of a finger-like and macro-void structure as a result of a fast solvent/non-solvent demixing during membrane formation.

In order to highlight the HNTs’ distribution within the polymeric PES matrix, which can impact on the performances of the membranes, the usage of a proton beam probe has been suggested and discussed in terms of the potentialities, as well as the main advantages, with respect to other common techniques. As reported in the Supplementary Information, preliminary tests have been performed on sub-micron unburied HNTs deposited on mylar, for comparing and associating AFM acquisitions to μ-PIXE maps measured over comparable scanning areas. The integrated interpretation of information obtained from μ-PIXE and FSTIM techniques has allowed us to map the presence and arrangement of HNTs over areas with extensions from 50 μm × 50 μm up to 1 mm × 1 mm, something which is not achievable using AFM.

Furthermore, it has been discussed how it is possible to obtain 2D FSTIM maps with volumetric information resulting from the different degree of scattering suffered by protons due to their interaction with matter and voids along the whole thickness of the membranes. Interestingly, this information has been demonstrated to be quantitatively consistent with that gained by means of the SEM cross-sectional imaging of the membranes. This point indicates a strong aspect of NM with respect to SEM, which suffers from the inconvenience of possible artefacts and impacts on the destructive preparation procedure.

Differently from SEM, which allows the investigation of only a few dozen nanometres in depth, due to the high scattering of the electron probe, a MeV proton beam, because of both its larger mass compared to the electron and its high acceleration, can pass deeper through the matter while retaining a straight line until the end of a range, depending on the proton energy and material.

The developed discussion has demonstrated the potentiality of the FSTIM technique for imaging the in-depth morphology of porous membranes, going beyond the information provided using SEM analysis because FSTIM maps also include information about the composition-related effective pore density through the proton scattering maps in a non-destructive way.

As far as we know, for the first time, NM has been used for the detection of the distribution of sub-micron HNTs embedded in polymeric membranes. We have also highlighted that the higher permeance of PES + [HNTs@PANI] (1%) is the result of a synergistic effect of PANI and HNTs, which foster an improved interplay of porosity, morphology, and mechanical properties within the composite membrane.

Definitively, NM was validated as a new method for the characterization of the composite membranes embedding HNTs, with the parallel evaluation of possible residual chemicals. We expect the technique can be used more generally for many different types of nanostructures integrating functional units with a wide range of compositions.

## Figures and Tables

**Table 1:** Thickness, water contact angle (WCA), pore size, porosity (calculated as reported in Equation (2)), mechanical properties, and water permeance (calculated as reported in Equation (1)) of the composite membranes PES, PES + PANI (1%), PES + HNTs (1%), and PES + [HNTs@PANI] (1%). See the experimental section for more details.

		Membrane (Dopant wt%)			
		PES	PES + PANI (1%)	PES + HNTs (1%)	PES + [HNTs@PANI] (1%)
Thickness (μm)		50	110	80	129
WCA (°)		70 ± 3	68 ± 1	69 ± 1	73 ± 3
Pore size (μm)		0.14 ± 0.01	0.52 ± 0.07	0.74 ± 0.01	1.91 ± 0.10
Porosity (%)		90 ± 1	86 ± 2	92 ± 1	89 ± 2
Mechanical properties	Young’s Modulus (MPa)	45 ± 6	54 ± 4	44 ± 3	43 ± 6
	Elongation at break (%)	4 ± 1	9 ± 2	7 ± 2	5 ± 2
Water permeance (L/m^2^ h bar)		1.8 ± 1.0	1.4 ± 0.5	1.6 ± 0.7	3.4 ± 0.5

**Table 2:** Percentage (in volume and density) of HNTs incorporated in the membranes PES + HNTs (1%) and PES + [HNTs@PANI] (1%) calculated via the ImageJ-based statistical analysis of the µ-PIXE maps acquired over areas of 50 µm × 50 µm, 100 µm × 100 µm, 500 µm × 500 µm, and 1 mm × 1 mm.

µ-PIXE Area	Volume (%)		Density (%)	
	PES + HNTs (1%)	PES + [HNTs@PANI] (1%)	PES + HNTs (1%)	PES + [HNTs@PANI] (1%)
1 mm × 1 mm	6%	5%	0.75	0.45
500 µm × 500 µm	6%	5%	0.75	0.45
100 µm × 100µm	5%	5%	0.62	0.45
50 µm × 50 µm	5%	4%	0.62	0.36

## Data Availability

The data that support the findings of this study are available on request from the corresponding authors, V. Arima, M. Carraro, and M. Cesaria.

## Supplementary Materials

The following supporting information can be downloaded at: https://www.mdpi.com/article/10.3390/nano13222970/s1, Scheme S1: preparation of the composite membrane PES + [HNTs@PANI], involving polymerization of PANI onto HNTs, dispersion of the resulting nanostructures in NMP, blending with PES dissolved in NMP, casting on a glass plate and immersion in a coagulation bath; Figure S1: Scheme of the combination of the PIXE-STIM-RBS techniques (a). Schematics of the STIM approach in the direct STIM mode (on the top) and forward STIM (b). Aluminum plaques with HNTs deposited on mylar (right) and with polymeric membranes (left) with 100 mesh copper grid fixed using silver paste (c); Figure S2: UV-VIS Absorbance of a HNT-PANI solution (0.2 mg/mL) in DMF; Figure S3: FTIR-ATR spectra of the composite (a) PES + PANI (1%) (b) PES + HNTs (1%) and (c) PES + [HNTs@PANI] (1%) membranes; Figure S4: WCA monitoring over time for (a) different PES membranes and (b) the composite PES + [HNTs@PANI] membranes. The star indicates that, at 140 s, the membrane completely absorbs water and swells. WCA measurements are quite difficult and WCA is supposed to be 0°; Table S1: Thickness, water contact angle (WCA), porosity, mechanical properties, and water permeance of the PES + [HNTs@PANI] membranes at different doping levels; Figure S5: SEM morphology of the investigated membranes: from the left to the right, top surface (5000×); bottom surface (5000×); cross-section (1000×); magnified cross-section (3000×); Figure S6: Two-dimensional AFM topographies of HNT distributions on Si at scanning areas (a) 20 μm × 20 μm, (b) 10 μm × 10 μm, (c) 5 μm × 5 μm, and (d) 2 μm × 2 μm showing the identification of a HNT sample in the distribution for decreasing scan areas. The yellow boxes represent the areas in which the next magnification scan was performed. For increasing magnification, single isolated HNTs can be clearly identified; Figure S7: (a) Two-dimensional AFM topography of HNT distributions on Si at scanning areas 30 μm × 30 μm. HNT distribution on mylar imaged by PIXE by scanning the proton beam over an area with an extension of (b) 30 μm × 30 μm (c) 250 μm × 250 μm and (d) 1mm × 1 mm. The detected elements are shown by the occurrence of aluminum (yellow) and silicon (green) signals. The red squares in panel (c) point out several 30 μm × 30 μm areas.
