# Supplementary material for: Polymeric Membranes Doped with Halloysite Nanotubes Imaged using Proton Microbeam Microscopy
Source: Nanomaterials (Basel). 2023 Nov 18;13(22):2970. doi: 10.3390/nano13222970 (PMC10674683; doi:10.3390/nano13222970)
Supplement: Supplementary file 1 [file nanomaterials-13-02970-s001.zip › nanomaterials-2711054-supplementary.pdf]

Supporting Information

Polymeric Membranes Doped with Halloysite Nanotubes Imaged using Proton Microbeam Microscopy

Giovanna Vasco [1,2], Valentina Arima [3,*], Soufiane Boudjelida [4,5], Mauro Carraro [4,6,*], Monica Bianco [3], Alessandra Zizzari [3], Elisabetta Perrone [3], Francesco Galiano [7], Alberto Figoli [7] and Maura Cesaria [2,*]

S.1 Material preparation

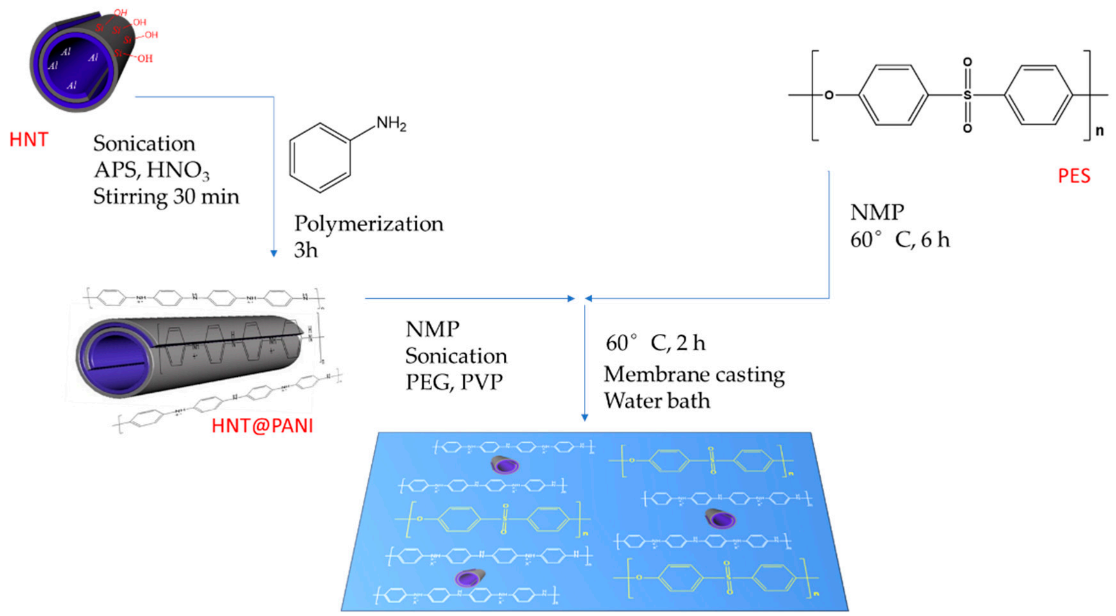

Scheme S1: preparation of the composite membrane PES+[HNTs@PANI], involving polymerization of PANI onto HNTs, dispersion of the resulting nanostructures in NMP, blending with PES dissolved in NMP, casting on a glass plate and immersion in a coagulation bath. HNT=halloysite nanotube, APS=ammonium persulfate, PES=Polyether sulfone, NMP=N-methyl-2-pyrrolidone, PEG=polyethylene glycol, PVP=polyvinylpyrrolidone.

S.2 PIXE and FSTIM set-up and analysis

Nuclear microscopy multi-elemental non-destructive investigation of both organic and inorganic materials was performed by the IBA (Ion Beam Analysis) nuclear microprobe beamline at CEDAD (Centre of Applied Physics, Dating and Diagnostics - University of Salento) [1,2], equipped to perform Proton Induced X-ray Emission (PIXE), Rutherford Back Scattering (RBS) and Scanning Transmission Ion Microscopy (STIM) based on a 3MeV energy proton beam probe accelerated by a 3 MV HVEE 4130HC Tandetron-type accelerator and focused by a triplet of high magnification low aberration quadrupole lenses (OM-150) by Oxford Microbeams Ltd allowing to obtain a proton beam spot size of 1-1.2 µm.

The available set-up lets perform simultaneously multi-elemental non-destructive investigations both on organic and inorganic materials. The system is equipped with a Si(Li) detector for X-rays (SSL30150 by Canberra), a Passivated Implanted Planar Silicon (PIPS) detector (ANPD300-19-100RM by Canberra) and a Si PIN photodiode (S1223 by Hamamatsu) in the vacuum chamber (OM-2000) to perform PIXE-RBS-STIM (Particle Induced X-ray Emission - Rutherford Backscattering

Spectrometry - Scanning Transmission Ion Microscopy) (Figure S1). Image detection was obtained by scanning the beam thanks to the pre-lens deflector coils (OM-25) connected to a dual channel power amplifier (OM-40e) and a staircase raster pattern generator (OM-30e).
In our investigation the analysis modes of PIXE and FSTIM were exploited.

A MeV proton going through a material interacts with both the atomic electrons and nuclei with a probability a few orders of magnitude larger for electrons than for nuclear scattering. The relevant mismatch in mass between protons and electrons implies that the trajectories of protons are straight-line paths, because of their being poorly affected by electron-proton interactions, and proton beams penetrate deeply into the material before coming to rest. Beam broadening occurs when the contribution of nuclear collisions become relevant.

PIXE is a non-destructive technique for multi-trace elemental analysis of the samples based on the detection of the characteristic X-ray emission caused by recombination events of the outer shell atomic electrons falling down to the inner-shell atomic-electron empty levels following MeV ion-atom interaction [3].

STIM detects the direct transmitted fraction of the probe particles interacting with a thin sample and generates spatial maps of the sample by measuring energy losses or variations in the number of the transmitted beam particles at each pixel of the recorded image. Instead of ion beams transmitted without significant angular scattering, small angle off-axis scattered transmitted ions can be detected by the so-called forward STIM (FSTIM) or off-axis STIM configuration. Such counting of the ions deflected at shallow angles with respect to the beam incidence axis can be useful to enhance the contrast of heavy elements with respect to low-mass element by exploiting the dependence of the cross section for nuclear elastic collisions on the squared atomic number Z_2 of the target element [4,5]. Therefore, in our experiments, the μ-PIXE technique allowed to obtain elemental information revealing the presence of the HNTs by the characteristic X-rays emitted by their elemental constituents following the interaction with a 3 MeV focused proton beam. The FSTIM technique provided density maps from the detection of the scattered transmitted protons throughout the sample with the detector positioned in the off-axis configuration at the deflection angle θ=20°.

For each sample, the μ-PIXE elemental information and the relative FSTIM maps were acquired over areas extending from 50 μm x 50 μm to 1 mm^2 by using beam currents lower than 1 fA operating for 1 hour. The optical microscope installed in the vacuum chamber (10^{-7} mbar) allowed both to translate two-dimensionally the samples along two independent directions and to control that no damage occurred. The data set was controlled by the OM_DAQ program.

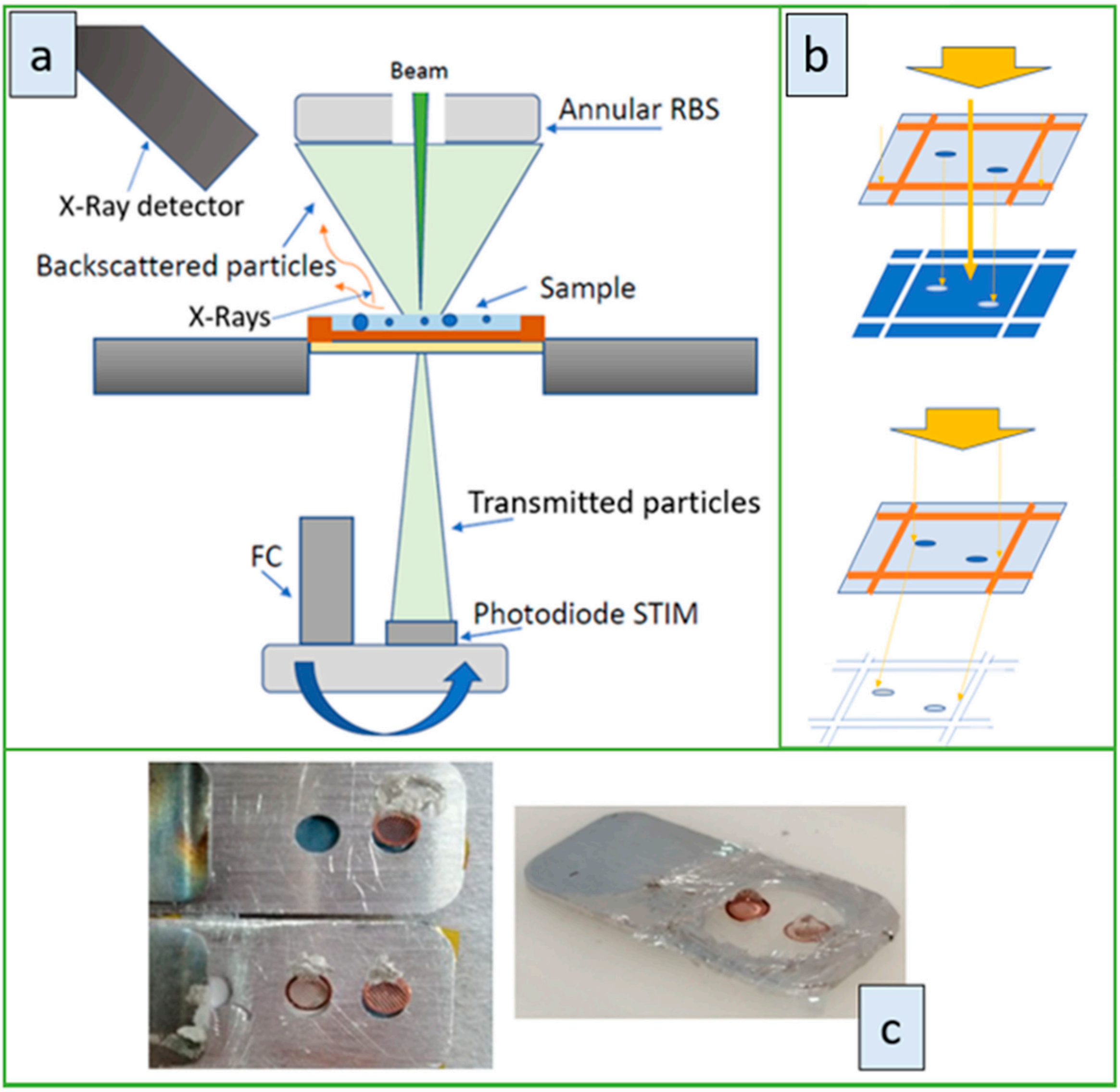

Figure S1: Scheme of the combination of the PIXE-STIM-RBS techniques (a). Schematics of the STIM approach in the direct STIM mode (on the top) and forward STIM (b). Aluminum plaques with HNTs deposited on mylar (right) and with polymeric membranes (left) with 100 mesh copper grid fixed by silver paste (c).

Despite the RBS technique allows the detection of light elements like C, N and O owing to the backscattered protons, in this study it was not possible to achieve a complete characterization because the working current of few fA, which is not sufficient for RBS analysis, is necessary to avoid damaging of the hydrated layer and halloysite shrinkage. Anyway, all of this is not a limit of our characterization because, in this context, we are interested in mapping the presence of HNTs trought their metallic constituents.

For NM analysis of HNTs, four samples were prepared by drop casting deposition of a droplet of 5 µl aqueous solution of HNTs (0.83 mg/mL in PBS 50 mM, pH 7.0) on a thin (0.9 µm thick) layer of mylar (a transparent polyester film made from stretched polyethylene terephthalate (PET)) placed over a perforated aluminium plaque to be attached to the NM sample holder for µ-PIXE and FSTIM measurements. Copper grids of 100 mesh were then applied on the sample surface to act as a spatial reference during proton beam irradiation of the samples (Figure S1(c)).

Turning to the membranes, two membranes with embedded HNTs (PES+HNTs and PES+PANI-HNTs, Table 1) and one membrane without HNTs (PES+PANI, Table 1) were then placed on the aluminium plaques of the NM sample holder with the copper grids (Figure S1(c)).

S.3 Spectral characterizations

The polymerization of PANI onto HNTs was monitored by UV-VIS upon dispersion of the sample in DMF, by using a Varian Cary 50 spectrophotometer, with 1 cm optical path quartz cuvettes, at T = 25°C. As Figure S2 shows, the peak at 800 nm, assigned to benzenoid-quinoid rings charge transfer, confirms the formation of the emeraldine salt (ES) form of polyaniline (conducting form). The bands at 350-420 nm are ascribed to π-π* transition of the amine benzenoid rings.

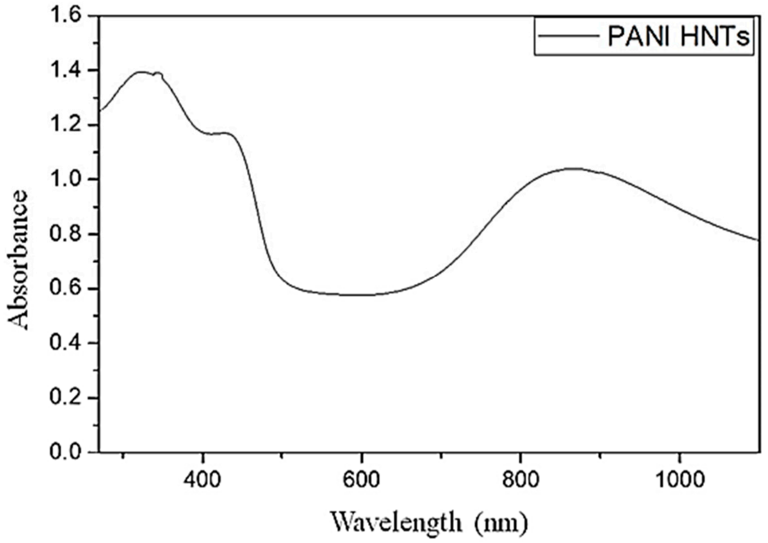

Figure S2: UV-VIS Absorbance of a HNT-PANI solution (0.2 mg/mL) in DMF.

FTIR-ATR spectra of the membranes under consideration in this study were obtained from solid samples using a Nicolet 5700 FT-IR instrument. As Figure S3 shows, PES signals are dominant in all cases. ATR-FTIR spectra of the membranes are basically unaffected by the small amount of dopants and show the expected signals pattern of PES matrix (Figures S3(a)-(c)) [6,7].

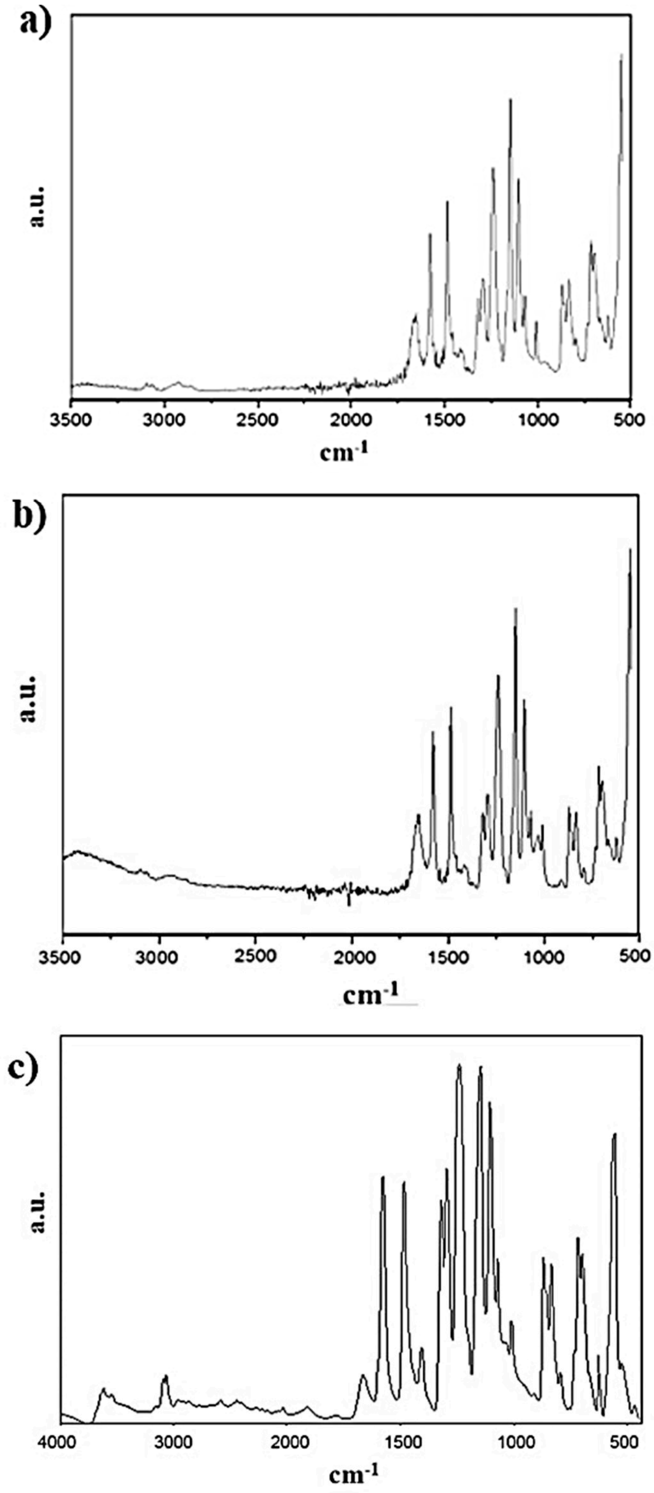

Figure S3: FTIR-ATR spectra of the composite (a) PES+PANI (1%) (b) PES+HNTs (1%) and (c) PES+ [HNTs@PANI] (1%) membranes.

S.4 Wettability characterizations

Water contact angles (WCAs) were measured by sessile drop method employing a CAM200 instrument (KSV Instrument LTD, Finland). The contact angle assays were repeated at different time points to evaluate the adsorption kinetics on the top surface of the membrane. Figure S4 shows the results of the WCA monitoring performed over time in the case of different PES membranes (Figure S4 (a)) and composite PES+ [HNTs@PANI] membranes (Figure S4 (b)).

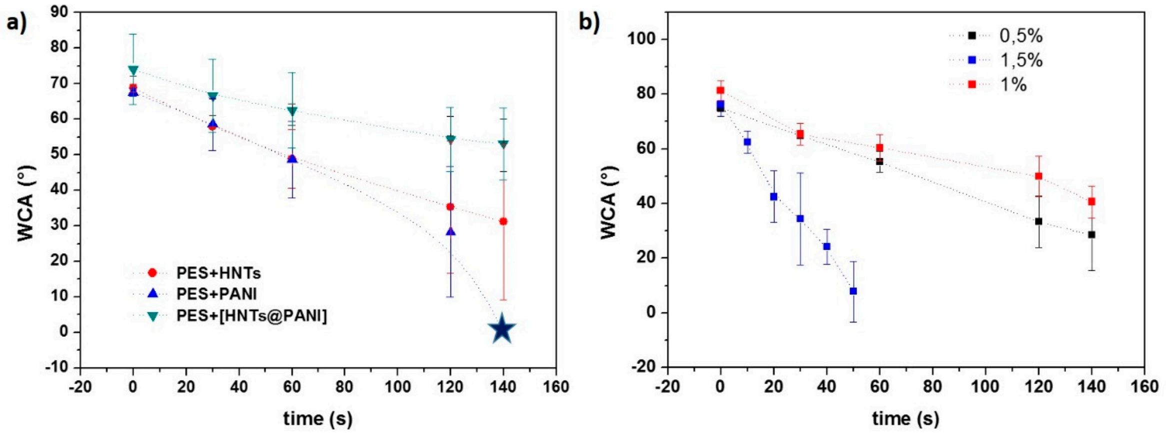

Figure S4: WCA monitoring over time for (a) different PES membranes and (b) the composite PES+ [HNTs@PANI] membranes. The star indicates that at 140s the membrane completely absorbs water and swells. WCA measurements are quite difficult and WCA is supposed to be 0°.

S.5 Additional characterizations on PES+[HNTs@PANI] at different dopant concentrations

Membranes with different content of HNTs were also investigated and characterized. The results of such investigations are reported in Table S1 and Figure S5.

Table S1. Thickness, water contact angle (WCA), porosity, mechanical properties, and water permeance of the PES+[HNTs@PANI] membranes at different doping levels.

Membrane (dopant wt%)	Thickness (µm)	WCA (°)	Pore size (µm)	Porosity (%)	Mechanical properties		Water permeance (L/m^2 h bar)
					Young's Modulus (MPa)	Elongation at break (%)	
PES+[HNTs@PANI] (0.5%)	100	73 ± 4	1.72 ± 0.4	93 ± 2	39 ± 4	8 ± 1	5.0 ± 2.3
PES+[HNTs@PANI] (1%)	129	73 ± 3	1.91 ± 0.1	89 ± 2	43 ± 6	5 ± 2	3.4 ± 0.5
PES+[HNTs@PANI] (1.5%)	116	71 ± 3	1.84 ± 0.3	87 ± 1	48 ± 4	7 ± 1	2.7 ± 0.1

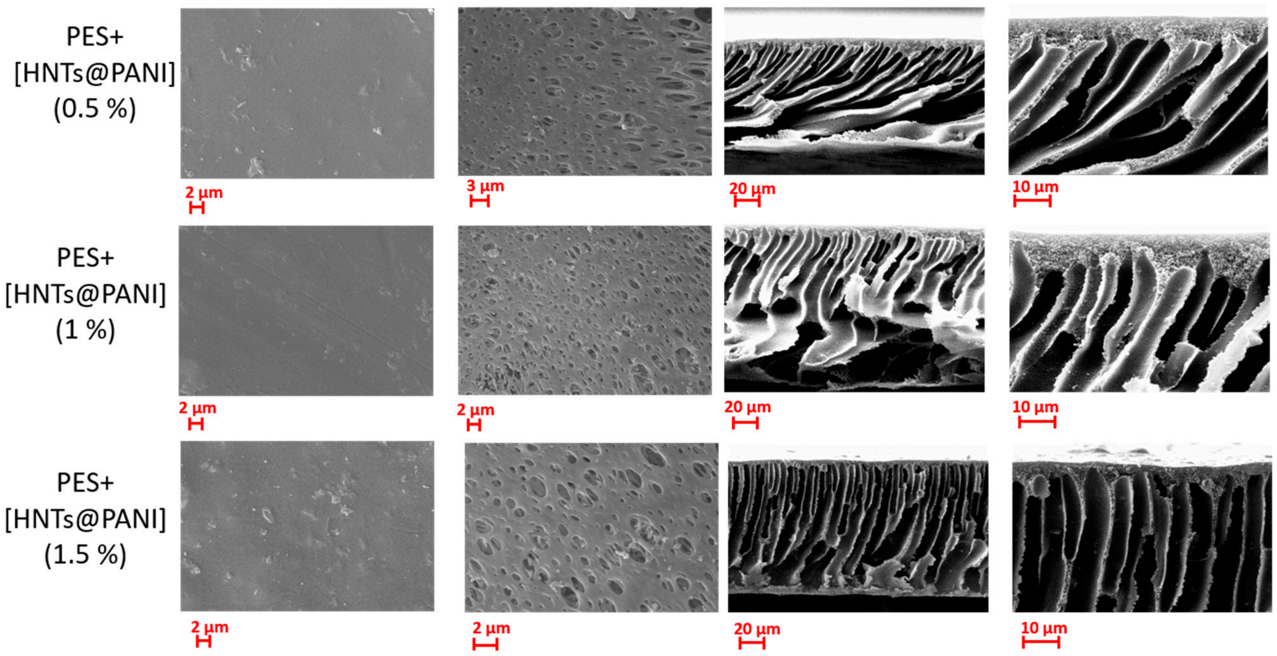

Figure S5: SEM morphology of the investigated membranes: from the left to the right, top surface (5000 x); bottom surface (5000 x); cross-section (1000 x); magnified cross-section (3000 x).

S.6 Comparison between AFM and NM investigation of HNT

Preliminary AFM analysis was performed for a few main purposes: first, to characterize the HNTs morphology, average size and surface features depending on functionalization and treatment steps through a standard technique; second, to point out the limits in the information that can be obtained routinely; third, to bridge the choice of the proton micro-beam probe not as an alternative but as a valuable, and in some aspects unique, investigation approach of spatially sub-micron sized HNTs.

Unlike standard techniques, AFM can be applied to a wide range of resolutions (from sub-micron to micron scales), requires no preparation of the sample and reduces cost and working time with respect to SEM and TEM.

Atomic force microscopy analysis discussed hereafter was performed for HNTs adsorbed on a silicon substrate. For NM analysis, HNTs were dispersed by drop casting deposition on a thin (0.9 µm thick) layer of mylar (see paragraph S2 and Figure S1). Due to the relevant differences in the substrate characteristics, no comparison in terms of distribution can be reliably drawn. This point is not a limit of our analysis because we aim at comparing the operative conditions and kind of information that can be obtained for the specific system of interest in this study.

Figures S6(a)-(d) show the sequence of AFM topography images acquired for decreasing scanning area from 20 µm x 20 µm to 2 µm x 2 µm, that is for increasing magnification, in such a way single HNTs and small agglomerated can be identified.

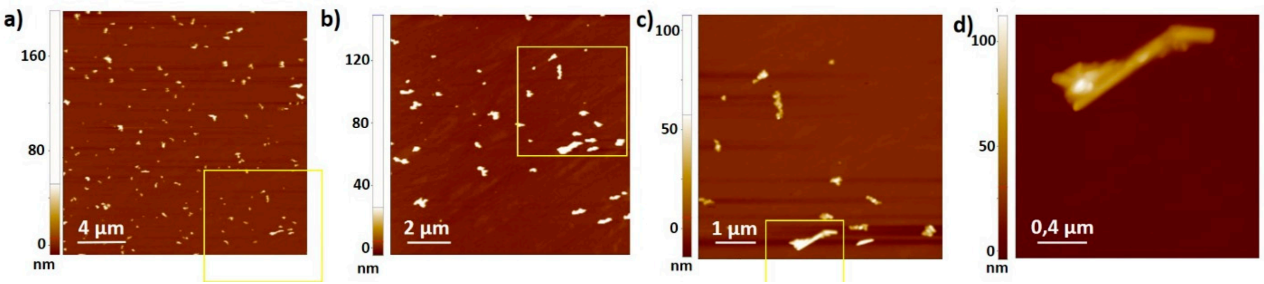

Figure S6: Two-dimensional AFM topographies of HNT distributions on Si acquired for decreasing scanning areas: (a) 20 µm x 20 µm, (b) 10 µm x10 µm, (c) 5 µm x5 µm, (d) 2 µm x 2 µm. The yellow boxes represent the areas in which the next magnification scan was performed. For increasing magnification, single isolated HNTs can be clearly identified.

At the maximum scanning area allowed by our AFM set-up (i.e., 30 x 30 µm, Figure S6(a)), no more than spatial arrangements and large aggregates of HNTs can be imaged, consistently with the submicron-scale size of the objects under consideration.

High-magnification AFM imaging conditions (Figures S6 (c), (d)) clearly allow to detect the presence of isolated HNTs and to get a hint of their size and shape. Also, Figure S6(d) highlights an elongated aggregate of HNTs with micrometric scale-length. Although HNTs are easily recognized, tip convolution effects influence the measurements. Noteworthy, a high density of structures is observed, which appear with a shape more rounded than the expected one. Artefacts due to a higher probability of tip contamination during its scanning over large areas of the sample cannot be ruled out and are likely responsible for such effects.

Figure S7 shows a comparison between a 30 µm x 30 µm extended AFM topography map of HNTs distributed on a Si substrate (Figure S7(a)) and HNTs distributed on a mylar layer imaged by PIXE over a 30 µm x 30 µm scanning area. Uneven distribution of the PIXE signal is due to roughness and inhomogeneity of the underlying mylar, caused by the difficulty in placing an easily breakable mylar membrane over any support. Micro-PIXE maps of HNT samples deposited on mylar were also aquired over scan size areas ranging from 50 µm x 50 µm up to 1 mm x 1 mm. In this respect, the maps over 250 µm x 250 µm and 1 mm x1 mm are shown in Figure S7(c) and S7 (d), respectively. Aluminum (Al) and silicon (Si) signals were detected, consistently with the composition of HNTs [8]. Noteworthy, the PIXE maps exhibiting major contribution from the Al signal (yellow signal) than from Si (green signal), despite the stoichiometry $Al_2(OH)_4Si_2O_5$ ratio of 1:1 of the HNTs, can be accounted for the spectrum shape. That is, since the Si peak has higher intensity than the Al one, the tail of the Si peak is shared with the selection of the detector channels related to the Al area.

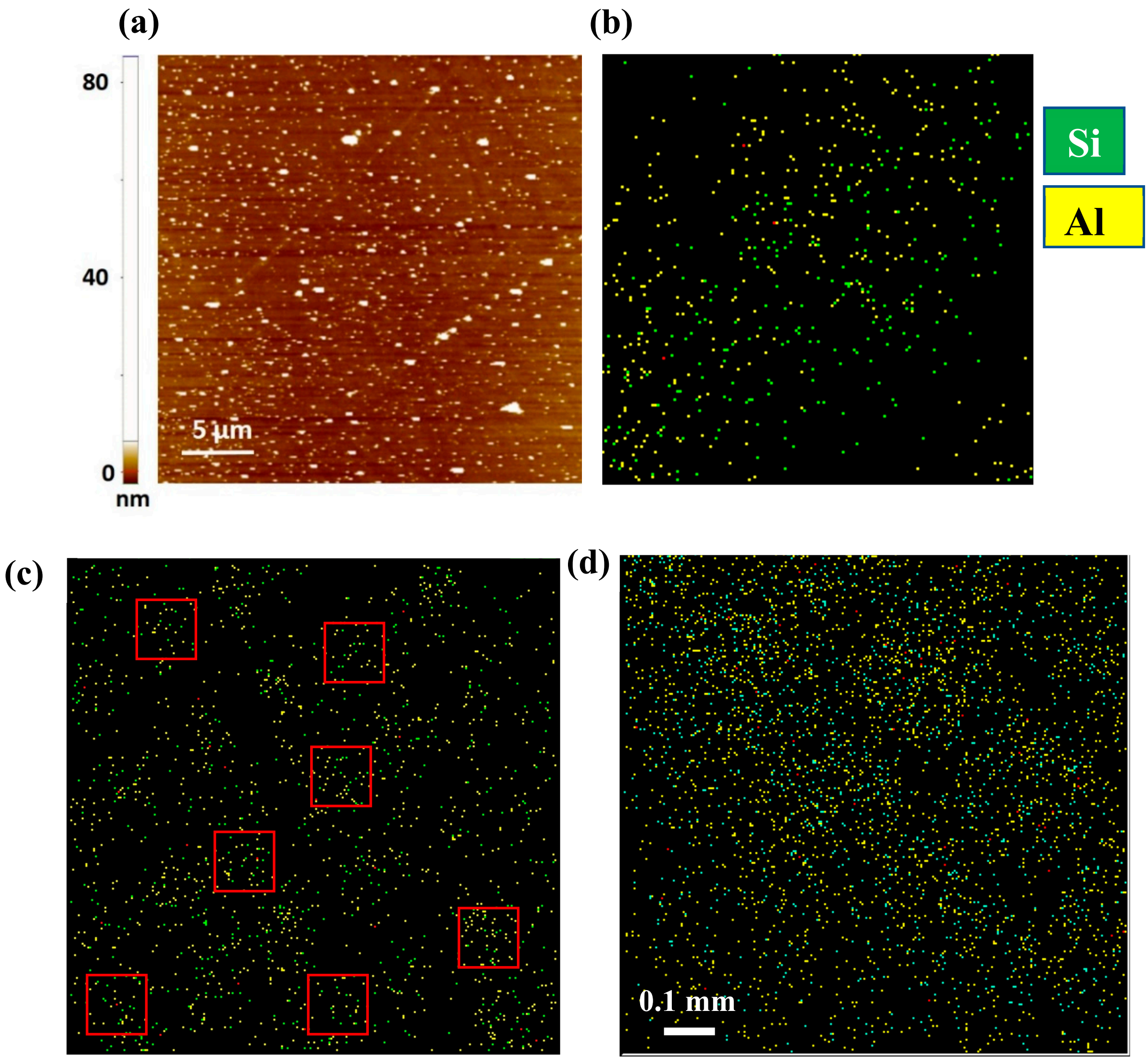

Figure S7: (a) Two-dimensional AFM topography of HNT distributions on Si at a scanning area of 30 μm x 30 μm. HNT distribution on mylar imaged by PIXE by scanning the proton beam over an area with extension of (b) 30 μm x 30 μm (c) 250 μm x 250 μm and (d) 1 mm x1 mm. The detected elements are shown by the occurrence of aluminium (yellow) and silicon (green) signals. The red squares in panel (c) point out several 30 μm x30 μm areas.

Consequently, since the photons counted in the selection are shown in the map of the relative element, a minimum part of the counts shown in the map of Al are actually related to the Si tail. Moreover, the higher intensity of the Si peak in the spectrum is in accordance with Garcia et al. 2017, where quantitative analysis by using X-Ray fluorescence (XRF) proved a slightly higher amount from Si than from Al [8]. The unpunctual correspondence between Al and Si depends mainly on the fluorescence yield and intensity as well as the occurrence of modes of deexcitation that are competitive with the emission of X-rays, such as the Auger effect.

Given the differences in the data acquisition as well as in the roughness and characteristics of the substrates onto which the HNTs are adsorbed (mylar versus silicon), AFM and NM techniques show a good agreement in the case of the scan area of 30 μm x 30 μm. Noteworthy, μ-PIXE is more convenient for scans over large areas because it is not influenced by tip artefacts and not limited by the AFM scanner technology. Indeed, the potential of the NM method is shown by μ-PIXE

acquisitions showing the HNT distribution on ever larger areas up to 1 mm^2 (Figure S7(d)). In the μ-PIXE map extending over 250 μm x 250 μm, red squares point out areas with extension of 30 μm x30 μm that can be exploited for statistical calculations without acquiring multiple images. Therefore, large area μ-PIXE scans let verify very easily the presence and characteristics of the distribution of HNTs as well as select region of interest for statistics.

Although NM doesn't allow to image the HNTs morphology at the sub-micron level, the comparison in Figure S7 points out the possibility offered by the NM technique to image the HNTs over areas comparable to the ones accessible by AFM without the drawbacks of AFM and, in addition, over areas of the order of mm^2 not accessible to AFM and electron microscopy.

References

1 Calcagnile, L.; Quarta, G.; D'Elia, M.; Muscogiuri, D.; Maruccio, L.; Butalag, K.; Gianfrate, G.; Sanapo, C.; Toma, U. Instrumental developments at the IBA-AMS dating facility at the University of Lecce. *Nucl Instrum Methods Phys Res B* **2005**, 240, 22–25.

2 Calcagnile, L.; Maruccio, L.; Scrimieri, L.; delle Side, D.; Braione, E.; D'Elia, M.; Quarta, G. Development and application of facilities at the Centre for Applied Physics, Dating and Diagnostics (CEDAD) at the University of Salento during the last 15 years. *Nucl Instrum Methods Phys Res B* **2019**, 456, 252–256.

3 Johansson, S. A. E.; Johansson, T. B. Analytical application of particle induced X-ray emission. *Nuclear Instruments and Methods* **1976**, 137, 473-516.

4 Chen, X.; Chen, C. B.; Udalagama, C. N. B.; Ren, M.; Fong, K. E.; Yung, L. Y. L.; Giorgia, P.; Bettiol, A. A.; Watt, F. High-resolution 3D imaging and quantification of gold nanoparticles in a whole cell using scanning transmission ion microscopy. *Biophys J* **2013**, 104,1419-25.

5 Lefevre, H. W.; Schofield, R. M. S.; Bench, G. S.; Legge, G. J. F. STIM with energy loss contrast: an imaging modality unique to MeV ions. *Nuclear Inst. and Methods in Physics Research, B* **1991**, 54, 363-370.

6 Noel Jacob, K.; Senthil Kumar, S.; Thanigaivelan, A.; Tarun M.; Mohan, D. Sulfonated polyethersulfone-based membranes for metal ion removal via a hybrid process. *J Mater Sci* **2014**, 49, 114–122.

7 Belfer, S.; Fainchtain, R.; Purinson, Y.; Kedem, O. Surface characterization by FTIR-ATR spectroscopy of polyethersulfone membranes-unmodified, modified and protein fouled. *J Memb Sci* **2000**, 172, 113–124.

8 Garcia-Garcia, D.; Ferri, J. M.; Ripoll, L.; Hidalgo, M.; Lopez-Martinez, J.; Balart, R. Characterization of selectively etched halloysite nanotubes by acid treatment. *Appl Surf Sci* **2017**, 422, 616-625.
